# Water rights shape crop yield and revenue volatility tradeoffs for adaptation in snow dependent systems

**DOI:** 10.1038/s41467-020-17219-z

**Published:** 2020-07-10

**Authors:** Keyvan Malek, Patrick Reed, Jennifer Adam, Tina Karimi, Michael Brady

**Affiliations:** 1 Department of Civil and Environmental Engineering, Cornell University, Ithaca, NY USA; 2 Department of Civil and Environmental Engineering, Washington State University, Pullman, WA USA; 3 School of Economic Sciences, Washington State University, Pullman, WA USA

**Keywords:** Hydrology, Agriculture

## Abstract

Irrigated agriculture in snow-dependent regions contributes significantly to global food production. This study quantifies the impacts of climate change on irrigated agriculture in the snow-dependent Yakima River Basin (YRB) in the Pacific Northwest United States. Here we show that increasingly severe droughts and temperature driven reductions in growing season significantly reduces expected annual agricultural productivity. The overall reduction in mean annual productivity also dampens interannual yield variability, limiting yield-driven revenue fluctuations. Our findings show that farmers who adapt to climate change by planting improved crop varieties may potentially increase their expected mean annaul productivity in an altered climate, but remain strongly vulnerable to irrigation water shortages that substantially increase interannual yield variability (i.e., increasing revenue volatility). Our results underscore the importance for crop adaptation strategies to simultaneously capture the biophysical effects of warming as well as the institutional controls on water availability.

## Abstract (web summary)

Studies on examining the climate impact on irrigated agriculture do not account for regional specific details. Here the authors studied both the direct and indirect impact of climate change on irrigated agriculture in the Yakima River Basin (YRB) and found that increasingly severe droughts and temperature driven reductions in growing season significantly reduces expected annual agricultural productivity.

## Introduction

Irrigated agriculture plays a crucial role in meeting global food demand. Although it uses only 20% of cultivated lands, nearly 50% of agricultural production^1^ relies on irrigation. The expansion of irrigated agriculture over the last 50 years is a significant contributor to increases in global food production that meet the needs of a growing population^2^. This historical trend is particularly important in light of the projected increases in food demand due to population growth and meat-intensive diets^3,4^.

Surface water resources account for 62% of the total worldwide water supply for irrigated agriculture. In many parts of the world, surface water supply depends heavily on snow accumulation and thawing regimes^5–7^. Most areas with latitudinal parallels over 40 degrees receive a majority of their precipitation in the form of snow^8^. There are also many mountainous regions in other latitudes that provide water for downstream agricultural regions^9,10^ including the Hindu Kush and Himalayas, the Karakoram, the Tien Shan massifs, the Andes, the mountains of the Middle East, and the Atlas Mountains^8^. Mountainous regions of the western United States (US), such as the Rockies, Cascades, Sierra Nevada mountains, are important snow-dependent regions^5,11,12^ that significantly influence national food production. In this study, we focus on the Yakima River Basin (YRB; Supplementary Fig. 1), an agriculturally important and heavily irrigated snow-dependent river basin^13,14^ in the Pacific Northwest of the US. Background on the YRB, including its regional context, and water rights structure can be found in the method section.

Climate change affects irrigated agriculture through two key mechanisms. First, the crops respond directly to climate change through biophysical mechanisms. Crop growth rate, productivity, and water demand will be affected by changes in atmospheric CO_2_ concentration, temperature, and precipitation (although for irrigated crops, precipitation plays a reduced role in affecting yield). While increasing CO_2_ concentrations increase yields (especially for crops with a C3 photosynthetic pathway), warming can deteriorate the productivity of some major crop types (especially annuals, such as corn, wheat, and potatoes) because of an acceleration in growth rate that also reduces the time available for photosynthesis and biomass accumulation^15–17^. There are many strategies that can be used to improve crop performance (average yield)^18,19^ including improvement in biotic resistance, abiotic resistance, and change in physical characteristics of crops. There is also a growing focus on slow-maturing crop varieties to reduce the impacts of temperature induced accelerated crop growth^16,18,20^. Although a broad body of literature has investigated this issue (readers can also see Supplementary Notes 3), the effectiveness of the yield-improving biophysical crop adaptations are not fully understood. The second mechanism where climate change impacts irrigated agriculture is the expected increase in the severity and frequency of droughts in snow-dependent areas^12–14^. In these basins, agriculture receives the majority of its summertime water supply from the melting of accumulated snowpacks. A warmer climate is expected to reduce snowfall and increase rainfall, reducing snowpack volume. In addition, higher temperatures accelerate the melting of snowpacks, further shifting water availability away from irrigation months^6,7,21,22^. This can lead to curtailment (or interruption) of water rights for irrigation, thus producing an indirect water scarcity effect from climate change on crop yields.

The emerging and potential future changes in the seasonality and magnitude of streamflow regimes in snow-dependent regions are unprecedented in their historical observations^11,13^. Therefore, characterizing candidate shifts in water availability requires modeling frameworks that account for regulated flows through managed storage facilities, institutional water rights, and important regulations^23^. All of these factors potentially limit the validity of many of the simulation-based assessments that do not account for these region-specific details.

Our research question is two-fold: first, What are the direct and indirect impacts of climate on irrigated agriculture in the YRB, and second, To what extent does farmer adaptation (through planting different crop varieties) aid adaptation against the negative effects of climate change? To address the first question, we investigate how the expected magnitude and interannual variability of yield in irrigated agriculture respond to the compounding (and sometimes competing) effects of changes in water availability, temperature, and atmospheric CO_2_ concentrations, and embedded within the water management and regulatory context of the YRB, a snowmelt-dependent watershed representative of many in the western US. To address the second, we explore how adaptive actions through crop variety improvements affect productivity in the YRB. In examining the impacts of both climate change and on farmer adaptation, we quantify the impacts to both expected mean yields, but also interannual yield variability, given the significant implications to crop insurance programs of the losses of revenue^24,25^ associated with extreme drought events. Our results show that climate change can impact the YRB by deteriorating its snow-driven water availability and accelerating crop biophysical processes leading to reduced average annual yields. We also show that in the absence of significant, simultaneous improvements in crop varieties, water governance institutions and the infrastructure of the YRB, adaptation efforts could face a significant tradeoff between improved average yields and increasingly severe revenue volatility in the region.

## Results

### Increasing water stress

**Fig. 1:**
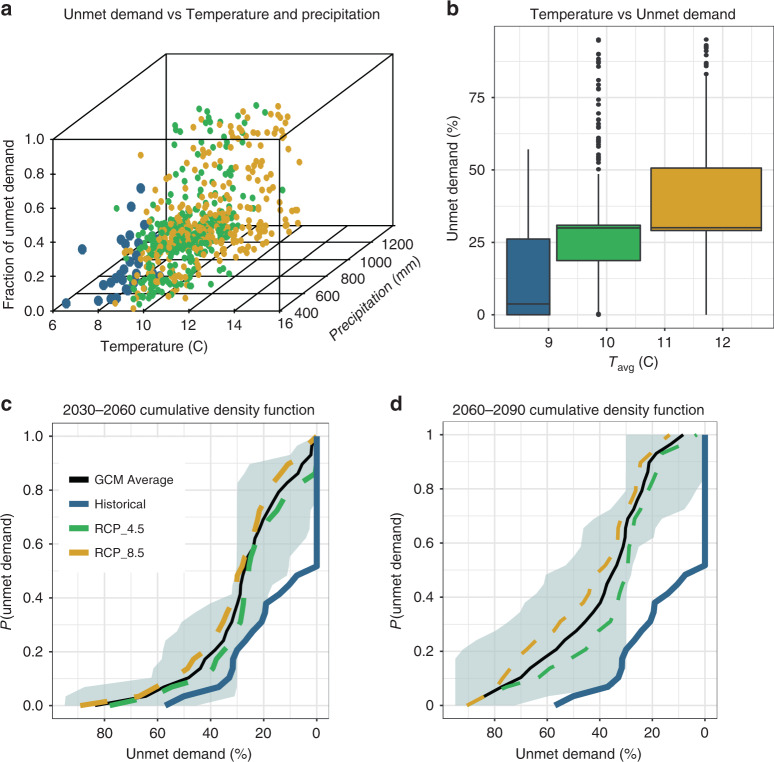
Impacts of climate change on water stress for irrigated agriculture. Panel **a** shows how unmet demand (UD) responds to temperature and precipitation. Each point corresponds to 1 year of simulation from one of the five GCMs used in this study. The historical period is from 1980–2010 and the future period is from 2030–2090. Panel **b** shows how unmet demand responds to temperature. In panels **b**, the interquartile range in the boxplots is 50% (lower and upper quartile limits are 25% and 75%, respectively). The middle line in the boxplot represents median, and the whiskers can span to 1.5 times the upper and lower interquartile ranges. Outliers (points) in this figure are numbers outside the whiskers. Also, in panel **b**, *n* = 300 simulated annual yield values for RCP 8.5 and 4.5 boxplots, and *n* = 30 simulated annual yield values for the historical boxplots. Panels **c** and **d** show cumulative density functions of the unmet demands for 2030–2060 and 2060–2090, respectively. Light shading in panels **d** and **e** represents the variation among GCMs and RCPs. Source data are provided as a Source Data file.

Irrigation unmet demand (UD, defined in the method section), an institutionally consistent drought indicator for the YRB, is strongly sensitive to climate change-induced changes in precipitation and temperature, showing transitions towards higher water stress (Fig. 1, panel a). This increase in UD is mainly controlled by temperature (Fig. 1, panel b) because the YRB, as a snow-dependent basin, receives 75–80% of its precipitation in the form of snow, and irrigation water availability in summer months depends heavily on the timing of snowmelt. Moreover, the active capacity of five major reservoirs in the YRB covers about 40% of total irrigation demand^26^. Therefore, the buffering capacity of this supply is not sufficient to compensate for this shift in streamflow timing. Moreover, as past studies^12,27^ have shown, in the Pacific Northwest, climate change does not produce a clear direction of change for precipitation, there is significant disagreement in the projections attained across the ensemble of available general circulation models (GCMs)^27,28^. Analysis of simulated future drought frequency (Fig. 1, panels c and d) also indicates that the probability of significant drought (UD > 30%) could significantly increase from 23% over the historical (1980–2010) period to ~40% [with a range of 13–63% across 5 GCMs  × 2 RCPs] over 2030–2060. During the same period (2030–2060), the average severity of significant droughts (UD > 30%) is projected to increase from historical UD of 40–46% of 6% relative to the historical average [with a UD projection range of 34–47% across the GCM model ensemble considered here]. Additionally, There there is the potential for unprecedented droughts in the YRB system (droughts stronger than with UD > 65%), which have not been recorded in the observed past. Our results show that for both time periods (2030–2060) and (2060–2090) there are even possibilities for experiencing very extreme droughts approaching the maximum value of water stress measure (UD = 100%).

### Agricultural productivity, yield, and interannual yield variability

**Fig. 2:**
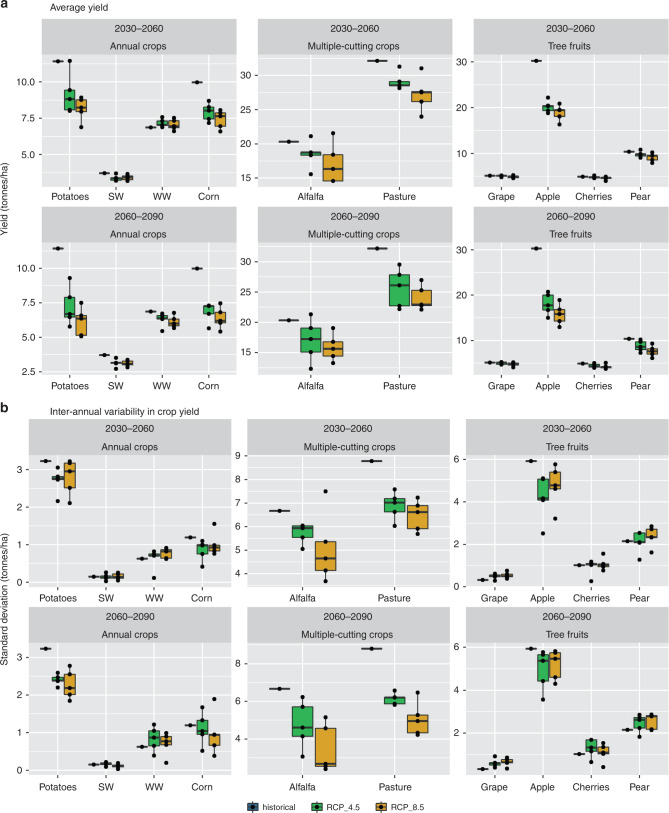
Future productivity of irrigated agriculture under simulated water shortage, precipitation, CO_2_ concentration, and temperature increases. Panel **a** shows average annual future yield and panel **b** shows standard deviation of annual agricultural yield. Crops have been categorized into three groups of annual crops (i.e., potatoes, winter wheat, spring wheat, and corn), multiple-cutting crops (i.e., alfalfa and pasture), and tree fruits (i.e., grapes, apples, cherries, and pears). Each panel has two rows that correspond to two different time periods: 2030–2060 and 2060–2090 (although the historical values are also included in both rows for comparison). The interquartile range in the boxplots is 50% (lower and upper quartile limits are 25% and 75%, respectively). The middle line in the boxplot represents the median, and the whiskers can span to 1.5 times the upper and lower interquartile ranges. Outliers (points) in this figure are numbers outside the whiskers. Also, in this figure, *n* = 10 30-year average and standard deviation of annual yield values for RCP 8.5 and 4.5 boxplots. Source data are provided as a Source Data file.

The ten most popular crops in the YRB are categorized into the following three groups (Fig. 2): first, annual crops (winter wheat, spring wheat, corn, and potatoes), second multiple-cutting crops (alfalfa and pasture), and third, tree fruits (grapes, apples, cherries, and pears). In terms of average agricultural productivity (Fig. 2, panel a and Supplementary Tables 3), all crop types show vulnerability to climate change with significant potential reductions in their average annual yields. For example, during the 2060–2090 period (RCP 8.5), average annual yield of potato, alfalfa, and apple could undergo a reduction of 46%, 22%, and 48%, respectively. This happens primarily due to the effects of more frequent and more severe droughts where shortages in available irrigation water occur. A portion of this reduction in the annual crop group can be attributed to a further reduction in maximum yield, which is defined as yield under fully irrigated condition (Supplementary Fig. 4, panel a and Supplementary Notes 5). The reduction in average yield of annual crops can cause damage to regional agricultural economies and even global food security because the deterioration of water availability and agricultural productivity follows the same trend across many snow-dependent regions of the world^5,6,10^.

During the 2030–2060 period (Supplementary Fig. 4), the maximum yield of annual crops such as spring wheat, corn, and potatoes show reductions of 9%, 23%, and 9%, respectively. These reductions result when higher temperatures accelerate the growth rates of the crops; in turn, causing earlier maturation and less opportunity for biomass accumulation^16,29^. However, we also show that the maximum yield of alfalfa could potentially increase in the future (26% during the 2030–2060 period). Alfalfa is a multiple-cutting crop, consequently faster growing cycles can increase the number of cuttings^30^ and enhance seasonal biomass accumulation. The minimum yield (under the no irrigation condition) increases for all crop types (Supplementary Fig. 4, panel b). For example over the 2030–2060 period, the minimum yield for spring wheat, corn, and potatoes increases by 17%, 25%, and 21%, respectively. Increased precipitation and CO_2_ concentration are the primary drivers of this improvement in non-irrigated yield^31,32^ and out-compete the yield-reducing impact of a shortened growing period. Consequently, the yield gap, which is defined here as the gap between the actual yield under water-stress conditions and the fully irrigated attainable yield, could decrease in the future. The reduction in yield gap has at least two significant implications: first, implications for Changes in the Marginal Benefit of Water (MBW): The shortened gap between fully irrigated and non-irrigated conditions also leads to lower absolute crop yield loss during drought years (Supplementary Fig. 3 and Supplementary Notes 4). The MBW in agriculture is defined as the magnitude of the improvement in yield in response to an extra unit of water^31^. The MBW has significant implications for investment decisions about new irrigation systems and other infrastructure^32^. A greater MBW indicates greater benefits for farmers who increase their water availability, thus making this investment more viable. Although we do not explicitly simulate MBWs, our results suggest that MBW will likely be lower in the future in YRB. When the yield gap is smaller (e.g., status quo crops under climate change scenarios), the MBW becomes smaller because an extra unit of water does not affect the productivity as much as in earlier periods. Therefore, annual crops undergo a reduction in MBW (e.g., 45% and 16% percent reductions in yield gap for corn and potatoes, respectively), but the MBW of multiple-cutting crops increases (e.g., an 11% increase in the alfalfa yield gap)**;** second, implications for Changes in the Interannual Variability of Yield: Although often overlooked in impact assessments of climate change, the interannual variability of yield is also important to consider; we show that although droughts are expected to intensify in the future, interannual variabilities of yield do not necessarily increase (Fig. 2, Panel b and Supplementary Table 3). Note that in our analysis, we use standard deviations (SDs) of annual series of yield to represent interannual variability of agricultural productivity. For example, throughout the 2030–2060 period, the reductions of SD for potatoes, corn, spring wheat, and alfalfa are 15%, 22%, 1%, and 19%, respectively. The primary explanation for these reductions in interannual variability is that maximum yield will decrease for many crop types (Supplementary Fig. 4, Panel a), including wheat, corn, and potatoes. Change in interannual variability of yield has direct implications for the parties involved in agricultural insurance programs, including insurance firms, insurance-buying farmers, and governmental insurance programs. Lower interannual variability of yield will result in higher accuracy in prediction of yield loss and, at the same time, fewer drought-related payouts to farmers^24,33^.

### Improved crop varieties

**Fig. 3:**
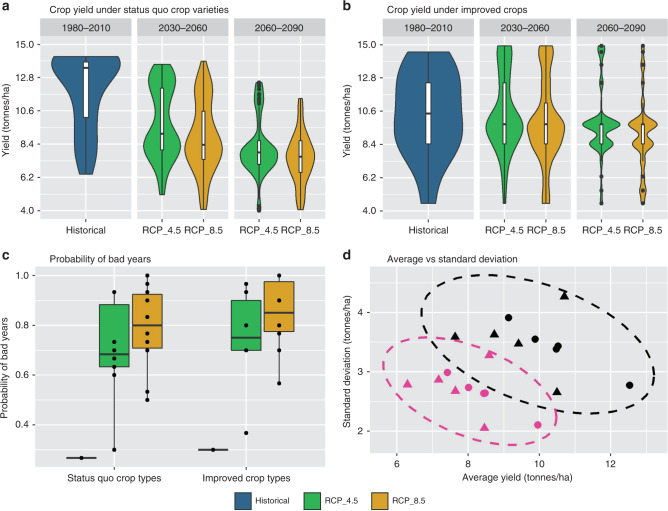
Effects of improved crop varieties on agricultural productivity of irrigated potatoes (see Supplementary Fig. 5–13 for other crops). Panel **a** shows status quo productivity of potatoes over the historical period (1980–2010) and the two future periods: 2030–2060 and 2060–2090. Panel **b** demonstrates how improved potato varieties affect yield. The plots in panels **a** and **b** are called violin plots, which are essentially inverted actual probability distribution plots. Inside the violin plots, there are boxplots with the interquartile range of 50% (lower and upper quartile limits are 25% and 75%, respectively). The middle line in the boxplot represents median, whiskers can span to 1.5 times of upper and lower interquartile range, and dots show outliers. Panel **c** shows how probability of bad years changes with new crop varieties; a bad year in this study is defined as years with productivity less than 60% of the fully irrigated yield. Boxplots in panel **c** have the same characteristics as boxplots inside the violin plots. Panel **d** shows the relationship between average yield and standard deviation for both the status quo crop (pink) and improved crop varieties (back). Oval curves in panel 4 show the bivariate Gaussian distribution for the confidence level of 0.95. Also, in panel **c**, *n* = 10 30-year average values of simulated yield calculated for five GCMs over two future periods. Source data are provided as a Source Data file.

As our future potential yield simulations indicate, the attainable yield of some important food-security crops could strongly decline (Fig. 2). This trend aligns with several previous studies’ findings and arguments^16,19,29,33,34^ that the use of current crop varieties can significantly reduce the productivity of some important crop types and potentially threaten global food security. In response to this issue, improvement in crop seeds has been proposed as a primary means of adapting to the adverse impacts of climate change on yields^19,20^. Improved varieties are expected to have longer growing periods to allow more time for photosynthesis and biomass accumulation^33^. In this study, we explore an adaptation scenario that considers seed-modification, and as demonstrated in Fig. 3 (Panels a and b), we show that during the 2030–2060 period, average yields of potatoes, spring wheat, and corn do improve with expected increases of 14%, 20%, and 31%, respectively (Supplementary Tables 2). However, for the same period (Supplementary Tables 3), the SD of yield is also shown to increase by 52%, 130%, and 123% for potatoes, spring wheat, and corn, respectively. Although these modified crops can compensate for the reduction in average annual potential yields, they remain vulnerable to deficits in irrigation availability during increasingly frequent and severe drought periods. In other words, average yield would increase, but at the same time, volatility in agricultural production and farmers’ revenue increases substantially (Fig. 3, Panel d). We also show that the probability of low-yield years (defined as years that produce under 60% of fully irrigated yield) may substantially increase with climate change (Fig. 3, Panel c). Although this trend is stronger under the RCP 8.5 scenario (Fig. 3, Panel c), the trend remains significant for a more optimistic RCP 4.5 future as well. Although we illustrate these trends using potatoes, similarresults occur for other crop types including multiple-cutting crops, tree fruits, and other annual crops (Supplementary Fig. 5–13). Higher variability in yield under improved crop varieties has uneven implications for insurance-buying farmers and governmental programs that subsidize crop insurance. Although more productive crop varieties enhance the volatility of the yield production, they will still increase average productivity, which expands the yield gap and increases the MBW. The greater MBW will make investment in agricultural water-supply projects (e.g., dams, water transfer projects, and irrigation systems) more likely.

### Insurance-related implications of changes in interannual variability

Our results on the relationship between climate change and yield variability has important implications for crop insurance programs^24^. At least two groups have a large stake in insurance programs: farmers and insurers. In many drought-sensitive areas, farmers sign up for insurance programs to reduce their losses when water scarcity causes low yields. Our results show that the current seed varieties for some important crops (e.g., wheat, corn, and potatoes), the potential for experiencing a very bad year (less than 60% of fully irrigated yield) declines. For farmers, overall agricultural revenue could be lower, but lower yield variability should mean reduced insurance premiums. Some governments, such as the U.S. federal government, subsidize insurance for certain crops^35^. Implications for these governments and other insurers, however, will likely be positive (in terms of the cost of such insurance programs), simply because the deviation from the normal condition and the perception of risk^36^ is lower. Therefore, we highlight that, in the absence of adaptation of seed varieties, the scenarios imply reductions in the cost of these insurance programs. Crop variety improvement, however, will reverse this because farmers could inadvertently become more prone to extreme low-yield years, and higher yield variabilities will likely enhance the necessity and cost of insurance programs for farmers^24,25,37^. In the US where the federal government subsidizes insurance primia for many crop types^36,38^, higher variability will likely increase governmental expenditure on insurance programs. Also, our results in this study imply that the relationship between yield loss and drought magnitude (e.g., Supplementary Fig. 3) will be fundamentally transformed under different climate change and crop variety improvement scenarios. These non-stationarities indicate the importance of actively reconsidering the underlying assumptions of index-based insurance programs^37^.

When contemplating the mean-variability tradeoff, and its winners and losers, it is important to consider who pays for the potential losses. In the US, there are post-Dustbowl programs that make the US federal government pay for the insurance of commodity crops (e.g., wheat, corn);^36,39^ therefore, the general public pays for the losses. However, there are many farmers in the US that purchase their own insurance^35^. In the case of insurance-buying farmers, because they pay for their own insurance, more volatility could increase their insurance premiums and reduce their annual revenue. It is also expected that the effects of this volatility would eventually be reflected in what consumers pay for their food^35^.

### Strategies to address the yield’s mean-variability tradeoffs

Our results imply that, in snow-dominated regions, two simultaneous strategies must be pursued to ensure a sustainable regional agricultural economy and productivity. First, water availability needs to be enhanced through improvements in water-related infrastructures and institutions. Examples of such strategies may include building water storage and transfer facilities, and adaptation of water rights and regulations. Second, improvement in potential productivity of crops. The results of our study emphasize the need for seed improvement strategies to carefully consider increasing sources of variability related to changes in temperature and irrigation water availability. To achieve that goal, it seems necessary to preserve the current diversity of seed varieties that have undergone different climatic conditions during several thousands of years^40^. Agrobiodiversity increases our ability to find and inject climate change-resilient varieties into the breeding process. In that regard, establishing efficient seed-sharing programs and banks^41^, promoting intellectual property rights platforms and agreements^42^, and embarking on deeper agriculture-related social and cultural studies^43^ seems to be the way forward. Also, strategies such as breeding under elevated temperature conditions hold promise for achieving yield improvement objectives^44^. In addition, faster plant-breeding processes can provide the research community with a higher degree of flexibility to create varieties that are resilient to elevated temperatures^19,44–46^. Although these production goals might be achievable, creating them could involve crucial hurdles. For example, past works^19,44^ have argued that, in order to efficiently create these high temperature-resilient varieties, the breeding process of these crops must become significantly faster. Currently, it takes about 30 years to create a new variety through conventional breeding; this will prevent us from making efficient and immediate adjustments in the breeding process. Moreover, based on the current trajectories of change, we might experience shortages of food before a breeding program can find an appropriate variety^44^. Moreover, recent research^45^ exploring different breeding experiments for wheat claim that our current genetic potential in production of temperature-resilient varieties might be limited due to the underlying gene availability. However, there are promising successes in developing temperature-resilient varieties^16,47^. Supplementary Fig. 2 approximately demonstrates how different water stress and crop variety scenarios may affect agricultural productivity (also, refer to Supplementary Notes 1 for more information). This figure underlines that, when crop varieties are improved without an improvement in the water system, there will be higher volatility in the overall agricultural production systems.

### Transferability of results to other snow-dominated regions of the world

The YRB is a heavily irrigated snow-dominated system, and our study (in agreement with prior studies^12,13,21^) shows that the system will undergo higher water stress as higher temperatures reduce snowpack. The other key pathway that negatively affects the YRB’s irrigated agriculture is the temperature-dependent reduction in potential yield^44,48^. We argue that, because both of these effects have been induced by higher temperatures, and higher temperatures are the most robust climate change effect that is expected to affect most of the planet^49,50^, our results could be of interest broadly to snow-dominated agricultural systems globally that must also manage the joint stressors institutionally constrained water availability and increasing temperatures.

## Discussion

In this study, we do not take into account other factors that might change in the future. These factors include a change in cropping pattern, shift in the geographical location of agricultural areas, and change in irrigation systems. Therefore, for example, 2060 does not realistically represent all of the factors that might be different in that year. Additionally, we do not take into account any possibility of change in current water rights and regulations. More specifically, scenarios that consider any possibility of imposing relinquishment are not included in our study. For example, under current western water law doctrine, long-term reduction in irrigation demand and diversion will be penalized by loss of water rights. However, relinquishment of unused water rights has not been historically practiced in the YRB. Finally, although the biophysical model used in this study to simulate crop heat stress, it does not capture crop mortality. Future work that captures a more refined simulation of crop heat stress and its dependence on the availability of irrigation water^51^ (used for cooling in addition to crop water demand) is warranted. Moreover, although beyond the scope of this work, a more realistic simulation of crop heat stress and its close dependence on the availability of irrigation water may be relevant to other studies. Such a simulation tool requires finer temporal resolution (hourly and shorter), tight coupling of an agro-hydrologic model with a regional atmospheric model, and a boundary-layer model that can accurately represent plant-level microclimates. Research initiatives in these directions can promote our understanding of crop response to higher temperatures, which is one of the most contentious aspects of future food production projections^15^. Additionally, in this study, we only consider one scenario of yield improvement (slower maturing varieties); however, there are many pathways that can alter the properties of crop production. For example, crops with shorter growing periods can be used as a drought escape strategy. They tend to reduce the risk of facing extreme water and temperature events and promise more reliable production. However, fast-growing varieties have less opportunity for yield accumulation and produce a lower amount of biomass and yield^48,52^. In other words, crops with shorter growing periods can introduce their own mean-variability tradeoff into the agricultural systems. A more in-depth exploration of possible seed-related adaptation strategies would be a beneficial extension of this study. Lastly, In this study, we do not take into account other factors that might change in the future. These factors include alterations in cropping pattern, shifts in the geographical location of agricultural areas, and changes in irrigation systems. Changes such as these could cause intended and unintended consequences that could propagate into different sectors and affect several aspects of the entire system. Therefore, our projections regarding 2060, for example, do not represent all of the candidate factors that might be shape yield dynamics in that year. However, the overall conclusions of this study emphasizing the need to simultaneously consider adaptation of seed varieties and their dependence on drought-related water institutions are broadly applicable across adaptation scenarios and different snow-dependent agricultural systems.

In summary, this study explores the effects of climate change on irrigated agriculture in the snow-dependent regions of the YRB. Modeling results show that there could be a significant increase of water stress on irrigated agriculture. This water stress and the shortening of growing seasons will reduce the overall productivity of agriculture. We argue that this overall reduction in productivity can substantially harm individual farmers, the regional economy, and, given the importance of irrigated agriculture in worldwide food production, even global food security. We also show that, although the uncertainty of water supply for irrigated agriculture increases in snow-dependent basins such as the YRB, the interannual variability of yield significantly decreases. We found this due to a concomitant reduction of the gap between fully irrigated crops and non-irrigated crops. Smaller yield gaps occur because the potential yield of some annual crops that are important to food security (such as winter wheat, corn, and potatoes) decreases. Put simply, we conclude that an increase in drought frequency and severity might not directly translate into more volatility in annual agricultural revenues. Furthermore, we explored a seed-modification adaptation, and we show that although it has the potential to improve overall agricultural productivity, the adaptation could lead to a significant increase in the revenue volatility of the food production system. This higher interannual variability can lead to challenges for insurers, governmental insurance subsidy programs, and insurance-buying farmers. Finally, we conclude that in order to ensure sustainable food productivity in irrigated regions, adaptation strategies should address both biophysical crop and water-supply considerations.

## Method

### Simulation tools

**Fig. 4:**
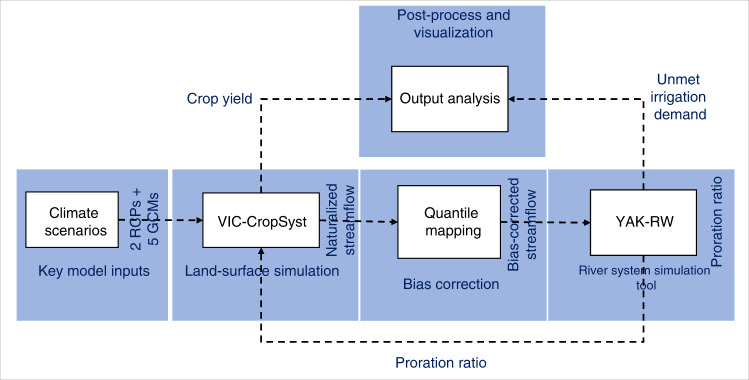
Simulation platform used in this study. Projected changes were attained using a tightly coupled agro-hydrologic land-surface model (VIC-CropSyst), quantile mapping-based bias correction, and the YAK-RW river system simulation. In this figure, dashed arrows indicate offline transfer of information between different components of the modeling platform.

We used a physically-based coupled agro-hydrologic platform that establishes connections between crop growth and development, land-surface hydrology, and river-system processes (Fig. 4). This framework has two main components: VIC-CropSyst^53^, which simulates agricultural and hydrological processes, and YAK-RW^54^ which is a river system (i.e., water management) model that simulates the operation of dams and prioritizes the allocation of water among different water-dependent sectors. The process-oriented and distributed nature of VIC-CropSyst makes our platform a powerful tool for capturing the effects of spatial variations in climate, soil, crop type, irrigation system, and topography. The capabilities of YAK-RW, on the other hand, allow us to reasonably simulate impacts of water rights and the operation of water facilities. A comprehensive description of all of the components of the simulation tools used in this study can be found in Malek et al.^14^.

Data: Simulation period and spatial resolution: The resolution of our simulation is 1/16 degrees. The historical period is from 1980 to 2010, and the future period is from 2030 to 2090. The following describes all of our model inputs.

Climate inputs: In this study, we provided minimum and maximum temperature, precipitation, and wind speed. The resolution of our meteorological data was 1/16 degrees. For the future period, we used outputs of five general circulation models (GCMs) forced by two representative concentration pathways (RCPs), RCP 4.5 and RCP 8.5, which are related to the radiative forcing of 4.5 and 8.5 $\frac{{w}}{{m^2}}$, respectively^55^. We used the method introduced by Brekke et al.^56^. to select these five GCMs from eighteen that were available. The GCMs used in this study include GFDL-ESM2G^57^, HadGEM2-ES365, HadGEM2-CC365^58^, INMCM4^59^, and CanESM2^60^.

Soil inputs: We used the same soil file as Malek et al.^53^. who used the State Soil Geographic dataset^61^ to develop soil inputs for the VIC-CropSyst.

Land cover information: We used the vegetation parameters file (VPF) and crop parameter file (CPF) used by Malek et al.^14^. The CPF includes information on crop acreage, planting date, irrigation system, crop type (perennial or annual), and proration rate. VPF provides the vegetation class, acreage, root depth, and root distribution in each soil layer.

Crop inputs: Some key crop-specific parameters of the VIC-CropSyst can be found in Supplementary Note 2 and Supplementary Table 1. These parameters are important for the simulation of agricultural processes such as growth, transpiration, biomass, and yield production. More comprehensive information on the crop input parameters can be found in the permanent repository of this paper; readers can also refer to Stockle et al.^62^ for more discussion of these parameters. Supplementary Note 3 also provides a thorough discussion of the assumptions involved in the development of the improved crop varieties considered in this study. Finally, Supplementary Note 6 and Supplementary Fig. 14 present a sensitivity analysis of these yield improvement assumptions.

### Yakima river basin

The YRB (Supplementary Fig. 1) is an intensively irrigated agricultural basin in central Washington State and spans an area of 16,000 square kilometers^63^. The basin-wide annual precipitation is about 680 mm, with the upstream mountainous regions receiving 2500 mm and the downstream regions receiving less than 200 mm. The YRB is a snowmelt-dominated basin^64,65^, and snow makes up 60–80% of its total precipitation^66^, Accumulated snow melts during the warm months (March to September) and feeds the Yakima River. There are five major dams in the YRB (Supplementary Fig. 1), which are capable of storing around 30% of the annual flow;^67^ they regulate streamflow and play a significant role in meeting irrigation demands. About 10% of employment in the area directly involves the agricultural sector, and irrigated agriculture generates more than $1 billion in annual revenue^63^. Yakima County is the state’s largest producer of many agricultural commodities (e.g., apples, grapes, and cherries).

### Water rights and water stress index in the Yakima river basin

Two types of water rights are provided by the U.S. Bureau of Reclamation’s Yakima Basin Project: (1) proratable and (2) non-proratable^26^. The irrigation water rights of proratable farmers can be curtailed during drought years when there is not enough water to meet agricultural demand. Non-proratable farmers, on the other hand, will always receive their full water rights, even in significant drought years. In YRB, a specific definition of water availability called proration rate is used to indicate the severity of a drought. In essence, proration rate is the ratio of water availability over water demand. More information on calculations of district-level proration rate can be found in Malek et al.^14,68^. In this study we use a more-universal term of unmet demand to represent drought severity. Fraction of unmet demand (UD) is defined as:

1 $${\mathrm{UD}} = 1 - \frac{{{\mathrm{Water}}\;{\mathrm{supply}}}}{{{\mathrm{Water}}\;{\mathrm{demand}}}}$$

Where, Higher UD indicate more water stress for farmers. Finally, in calculation of future basin-wide UD we took the reduction of irrigation of demand into account.

### Reporting summary

Further information on research design is available in the Nature Research Reporting Summary linked to this article.

## Supplementary information

Supplementary Information

Reporting Summary

## Data Availability

Although the supplemental materials provide a comprehensive description of the data and models used in this study, readers can refer to the GitHub repository of this paper to access the model, data and our post-processing scripts (10.5281/zenodo.3743865). Source data are provided with this paper.

## Source data

Source Data

## Code availability

Readers can refer to the GitHub repository and Digital Object Identifier (10.5281/zenodo.3743865) to access the scripts used to generate the results of this study. Source data are provided with this paper.
