# Supplementary material for: Water rights shape crop yield and revenue volatility tradeoffs for adaptation in snow dependent systems
Source: Nat Commun. 2020 Jul 10;11:3473. doi: 10.1038/s41467-020-17219-z (PMC7351950; doi:10.1038/s41467-020-17219-z)
Supplement: Supplementary file 1 — Supplementary Information [file 41467_2020_17219_MOESM1_ESM.pdf]

Supplementary Information:

Water Rights Shape Crop Yield and Revenue Volatility Tradeoffs for Adaptation in Snow Dependent Systems

Malek et al.

Supplementary Figures

Supplementary Figure 1

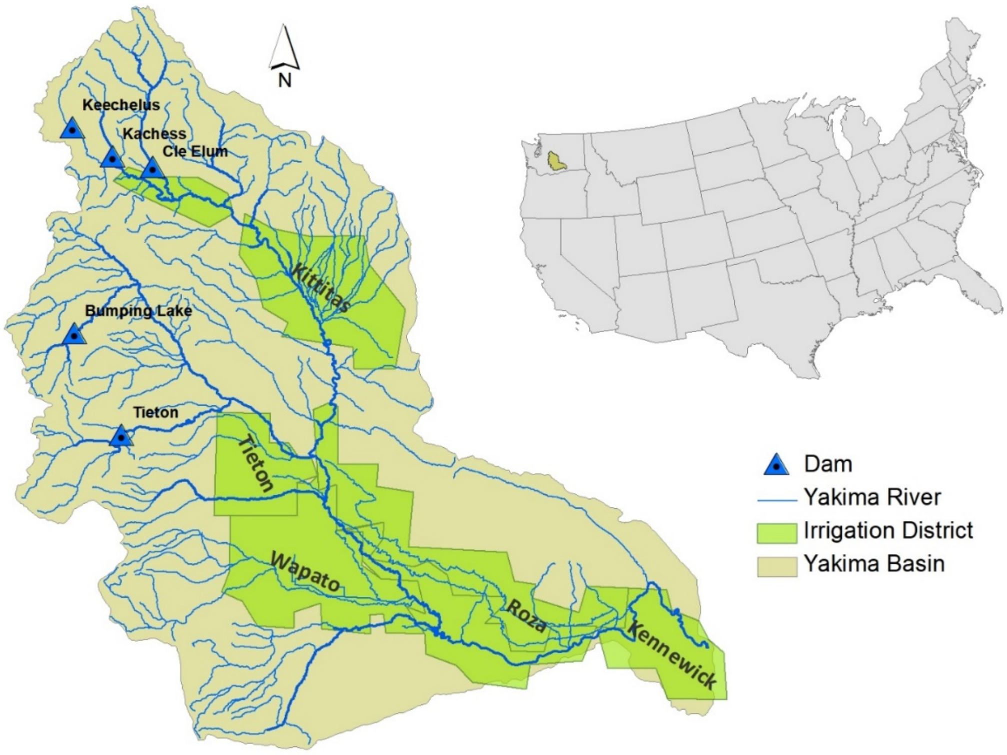

Supplementary Figure 1 - The Yakima River Basin is in central Washington State, U.S. (see inset). The figure includes the location of Yakima River and its tributaries, five major dams of the Yakima Basin, and six main irrigation districts in the region.

Supplementary Figure 2

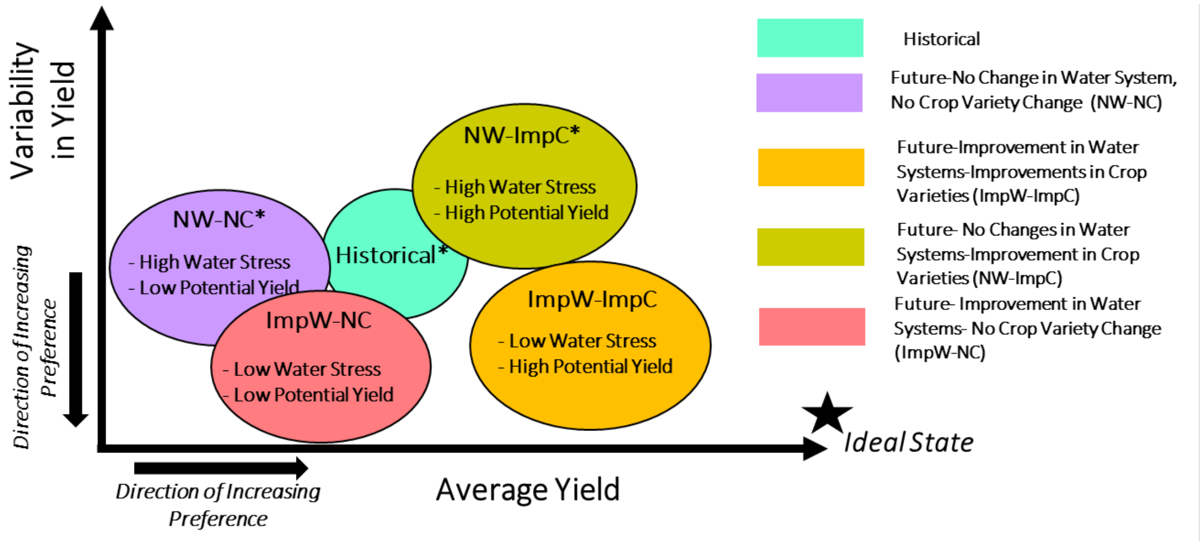

Supplementary Figure 2 - Impacts of climate change and water- and crop- related adaptation strategies on yield and revenue volatility tradeoffs. Although this diagram is conceptual and the details of it are not based on observations/simulations, asterisked (*) scenarios have been already simulated and presented in this study. In this figure, lower water stress can be achieved by improving water institutions and infrastructures and higher potential yield can be obtained through improvement in crop varieties. More discussion on this topic can be found in Supplementary Notes 1.

Supplementary Figure 3

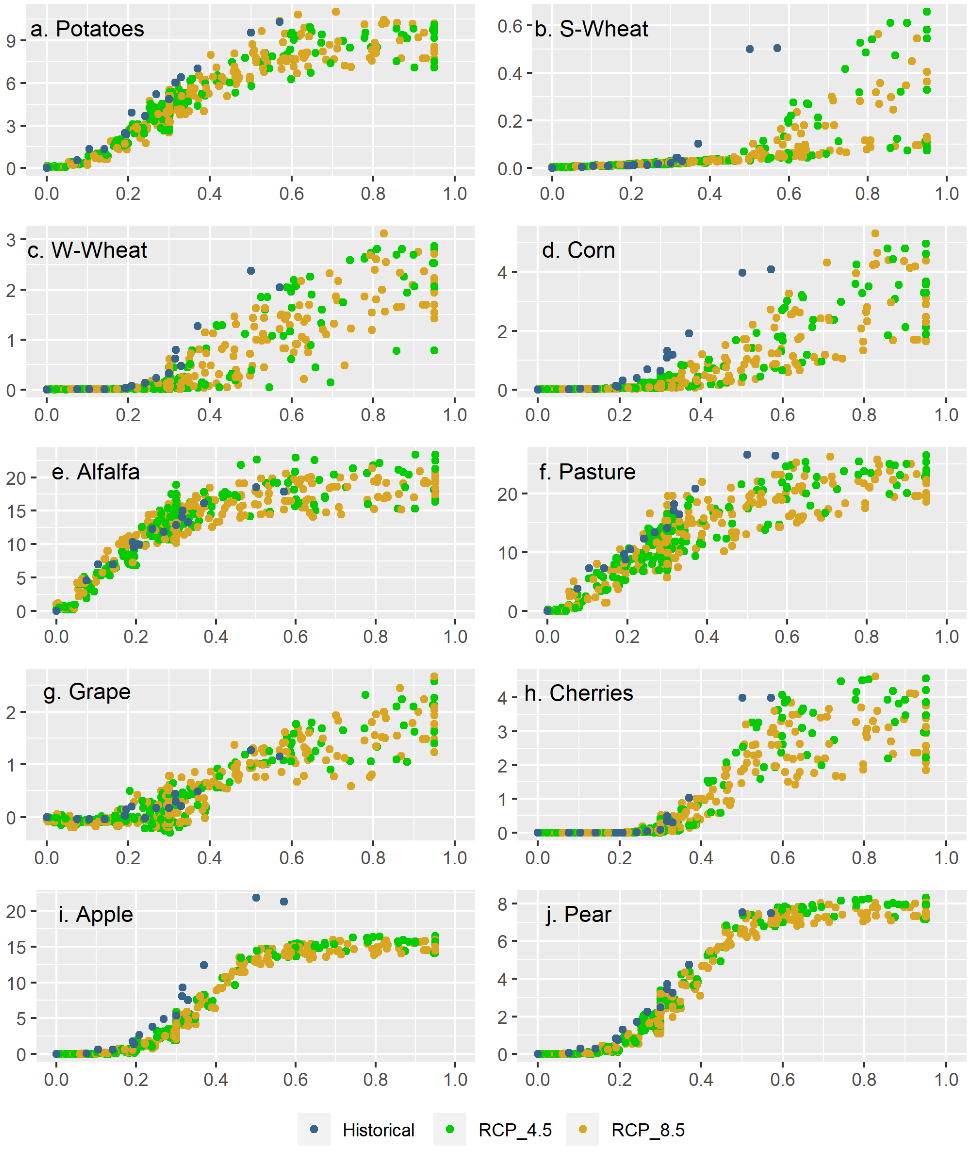

Supplementary Figure 3 - Yield loss defined as difference between crop yield under fully irrigated condition and yield under simulated unmet demand. Each point corresponds to one year of simulation from one of the five GCMs used in this study. Historical period is from 1980-2010 and future period is from 2030-2090.

Supplementary Figure 4

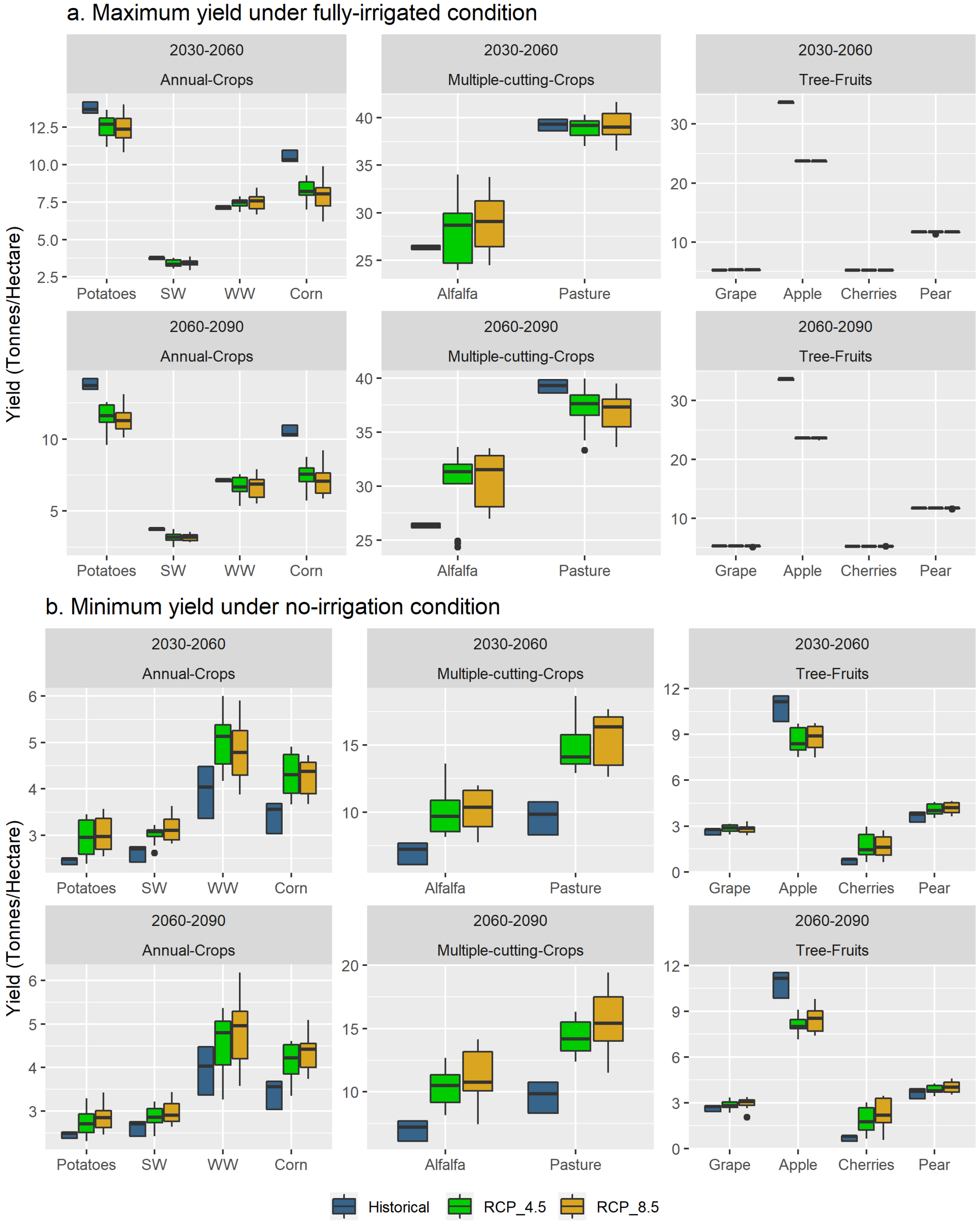

Supplementary Figure 4 - Maximum and minimum yield. Panel a. shows maximum yield defined as yield under fully irrigated condition. Panel b. shows minimum yield defined as yield under non-irrigated condition. Crops have been categorized into three groups of annual crops (i.e., potatoes, winter wheat, spring wheat, and corn), multiple cutting crops (i.e., alfalfa and pasture) and tree fruits (grapes, apple, cherries and pear). Each panel has two rows that corresponds to two different time periods of 2030-2060 and 2060-2090. In this figure, the interquartile range in the boxplots is 50% (lower and upper quartile limits are 25% and 75%, respectively). The middle line in the boxplot represents median, and the whiskers can span to 1.5 times the upper and lower interquartile ranges. Outliers (points) in this figure are numbers outside the whiskers. Also, in this figure, n=150 simulated yield values for RCP 8.5 and 4.5 boxplots, and n=30 simulated yield values for historical boxplots.

Supplementary Figure 5

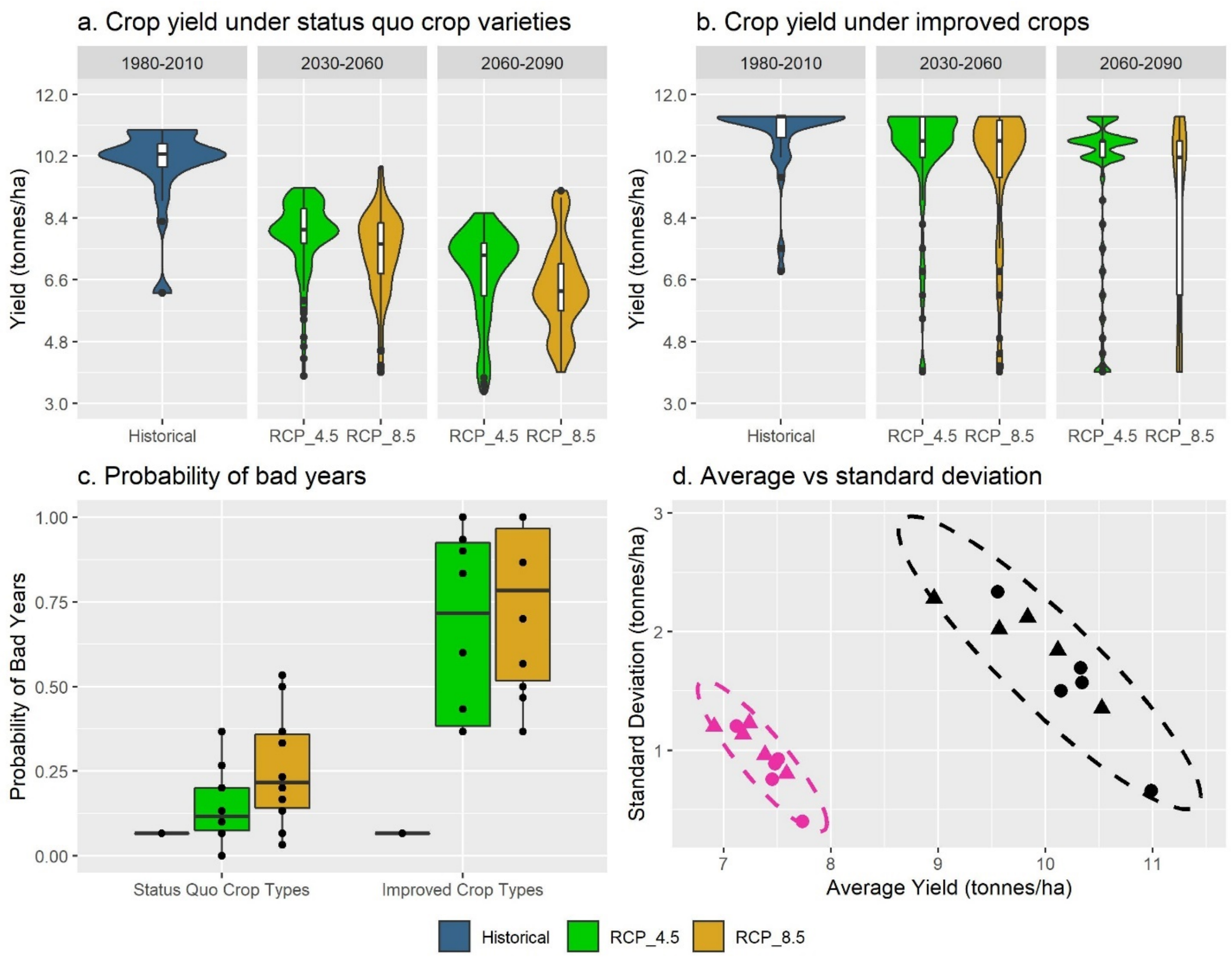

Supplementary Figure 5 - Effects of improved crop varieties on agricultural productivity of irrigated corn. Panel a shows status quo productivity of corn over the historical period (1980-2010) and the two future periods: 2030-2060 and 2060-2090. Panel b demonstrates how improved corn varieties affect yield. Panel c shows how probability of bad years changes with new crop varieties, a low yield year in this study is defined as years with productivity less than 60% of fully irrigated condition. Panel d shows the relationship between average yield and standard deviations assuming an unchanged status quo and improved crop varieties. In this figure, the interquartile range in the boxplots is 50% (lower and upper quartile limits are 25% and 75%, respectively). The middle line in the boxplot represents median, and the whiskers can span to 1.5 times the upper and lower interquartile ranges. Outliers (points) in this figure are numbers outside the whiskers. Also, in Panel c, n=10 30-year average values of simulated yield calculated for five GCMs over two future periods.

Supplementary Figure 6

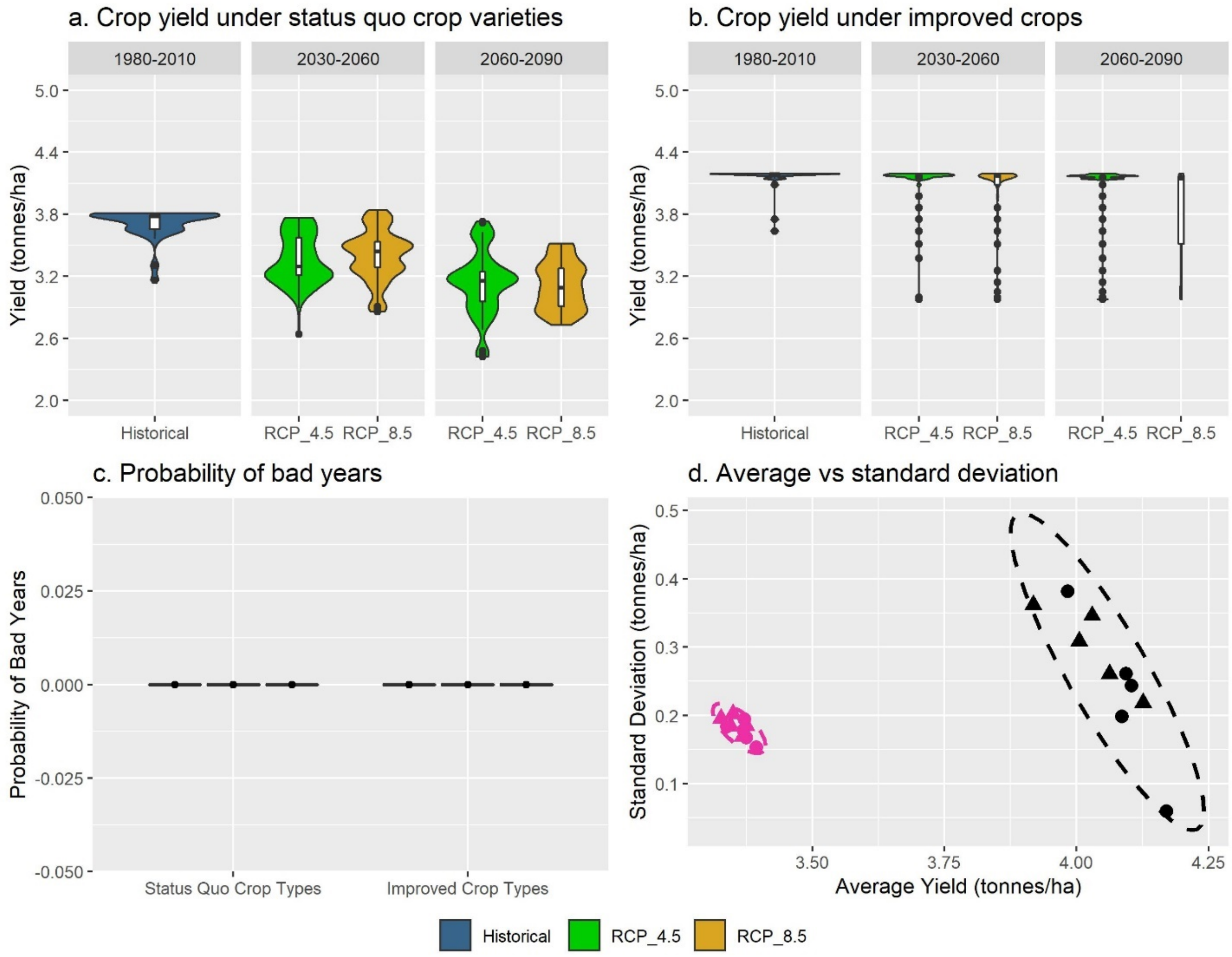

Supplementary Figure 6 - Effects of improved crop varieties on agricultural productivity of irrigated spring wheat. Panel a shows status quo productivity of spring wheat over the historical period (1980-2010) and the two future periods: 2030-2060 and 2060-2090. Panel b demonstrates how improved spring wheat varieties affect yield. Panel c shows how probability of bad years changes with new crop varieties, a low yield year in this study is defined as years with productivity less than 80% of fully irrigated condition. Panel d shows the relationship between average yield and standard deviations assuming an unchanged status quo and improved crop varieties. In this figure, the interquartile range in the boxplots is 50% (lower and upper quartile limits are 25% and 75%, respectively). The middle line in the boxplot represents median, and the whiskers can span to 1.5 times the upper and lower interquartile ranges. Outliers (points) in this figure are numbers outside the whiskers. Also, in Panel c, n=10 30-year average values of simulated yield calculated for five GCMs over two future periods.

Supplementary Figure 7

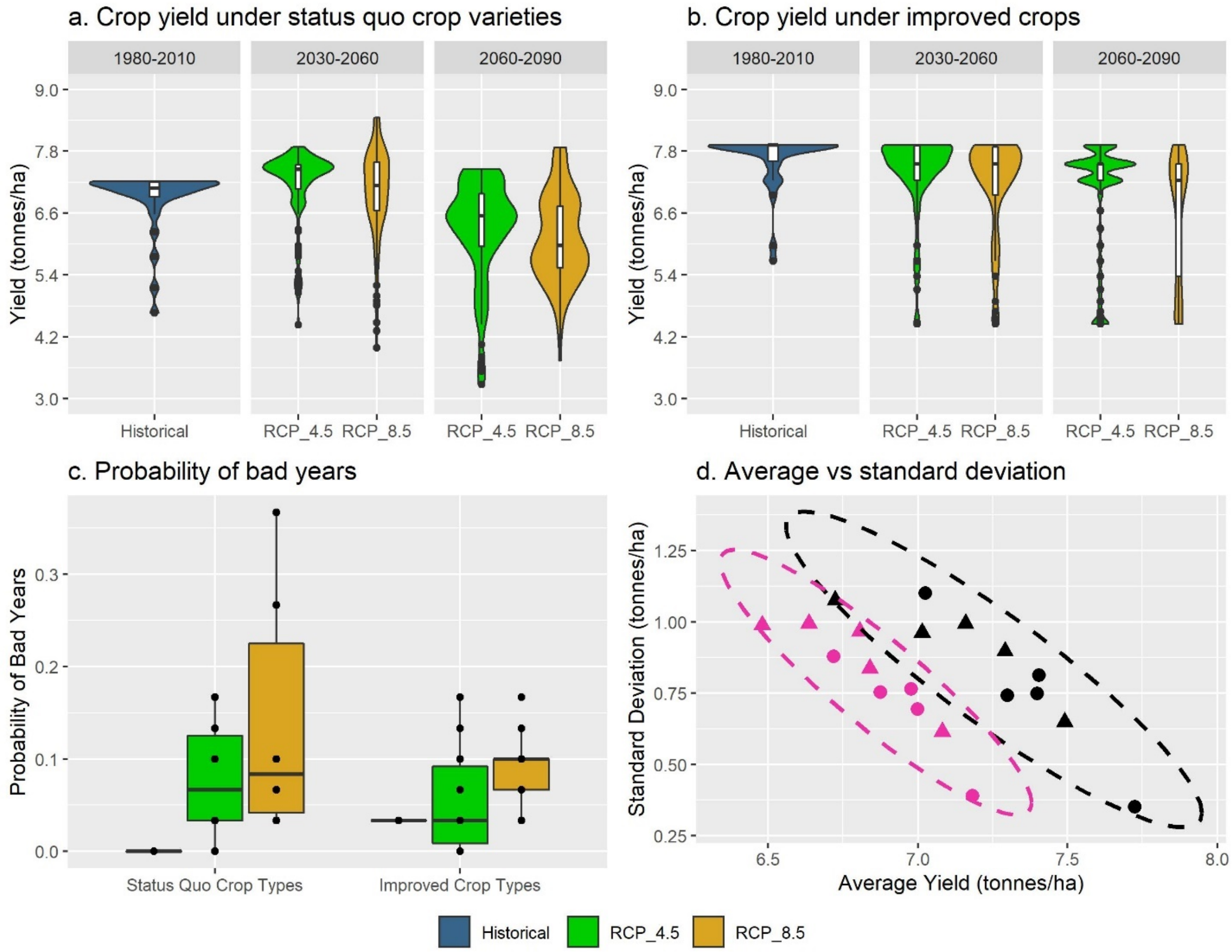

Supplementary Figure 7 - Effects of improved crop varieties on agricultural productivity of irrigated winter wheat. Panel a shows status quo productivity of winter wheat over the historical period (1980-2010) and the two future periods: 2030-2060 and 2060-2090. Panel b demonstrates how improved winter wheat varieties affect yield. Panel c shows how probability of bad years changes with new crop varieties, a low yield year in this study is defined as years with productivity less than 80% of fully irrigated condition. Panel d shows the relationship between average yield and standard deviations assuming an unchanged status quo and improved crop varieties. In this figure, the interquartile range in the boxplots is 50% (lower and upper quartile limits are 25% and 75%, respectively). The middle line in the boxplot represents median, and the whiskers can span to 1.5 times the upper and lower interquartile ranges. Outliers (points) in this figure are numbers outside the whiskers. Also, in Panel c, n=10 30-year average values of simulated yield calculated for five GCMs over two future periods.

Supplementary Figure 7

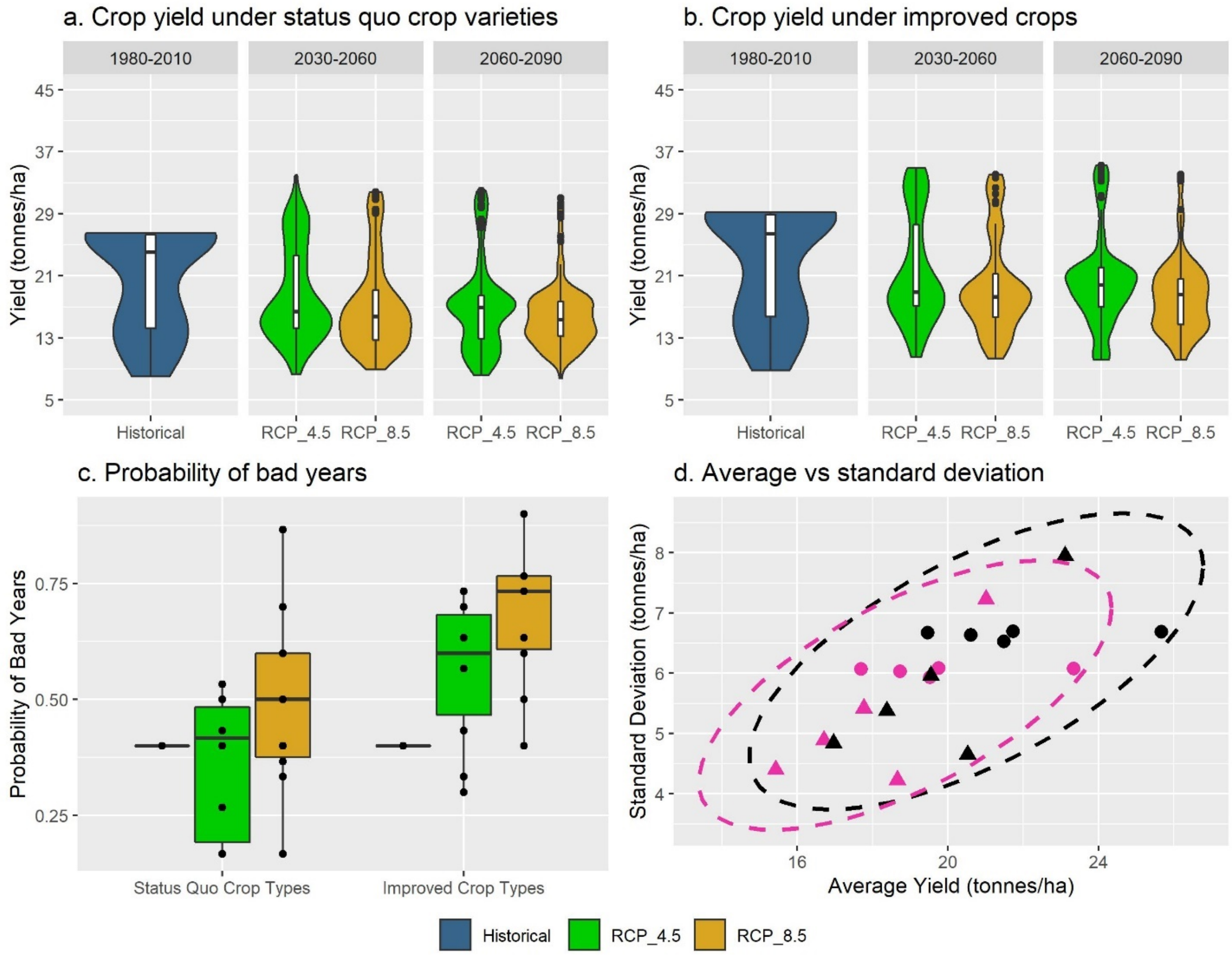

Supplementary Figure 8 - Effects of improved crop varieties on agricultural productivity of irrigated alfalfa. Panel a shows status quo productivity of alfalfa over the historical period (1980-2010) and the two future periods: 2030-2060 and 2060-2090. Panel b demonstrates how improved alfalfa varieties affect yield. Panel c shows how probability of bad years changes with new crop varieties, a low yield year in this study is defined as years with productivity less than 60% of fully irrigated condition. Panel d shows the relationship between average yield and standard deviations assuming an unchanged status quo and improved crop varieties. In this figure, the interquartile range in the boxplots is 50% (lower and upper quartile limits are 25% and 75%, respectively). The middle line in the boxplot represents median, and the whiskers can span to 1.5 times the upper and lower interquartile ranges. Outliers (points) in this figure are numbers outside the whiskers. Also, in Panel c, n=10 30-year average values of simulated yield calculated for five GCMs over two future periods.

Supplementary Figure 8

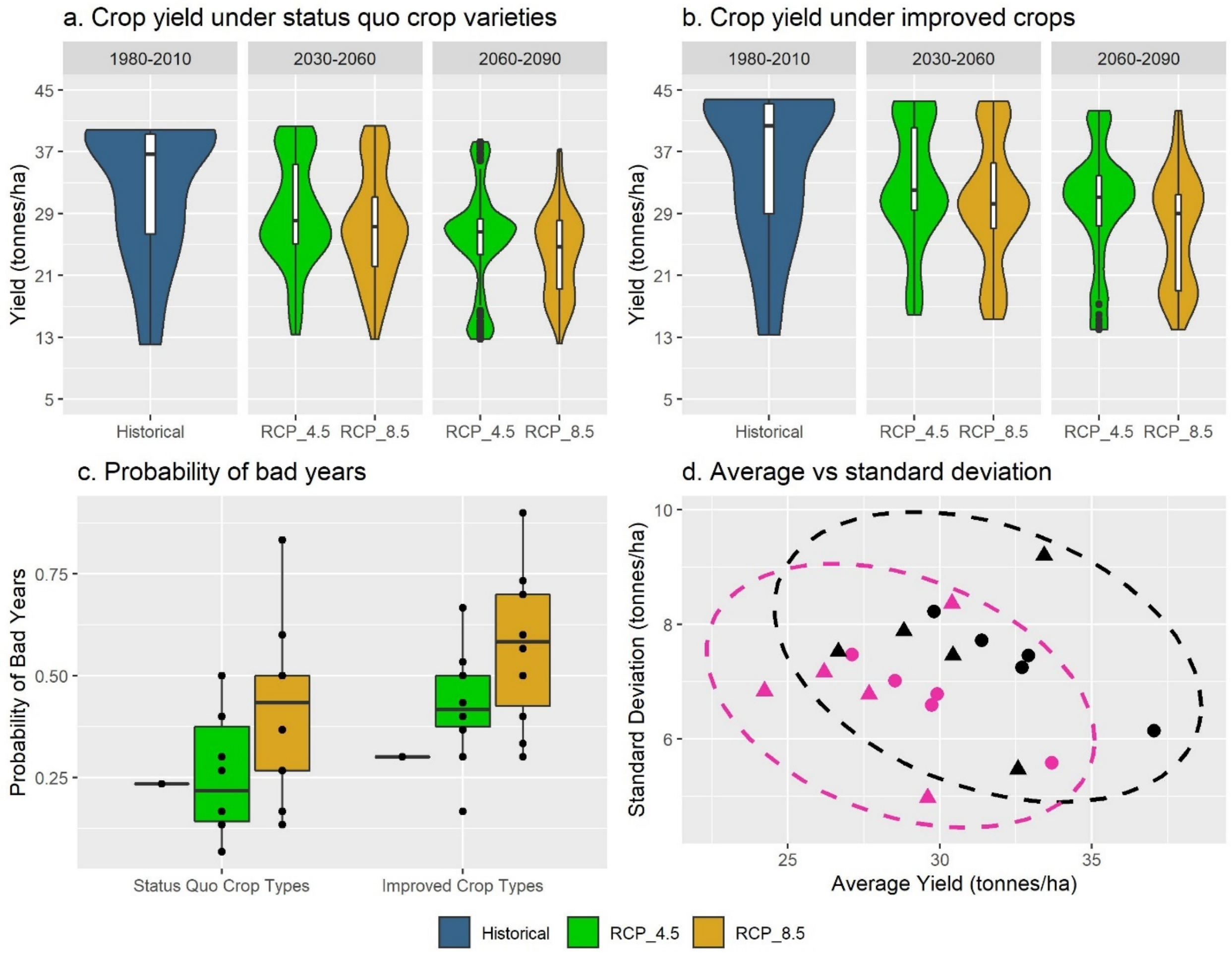

Supplementary Figure 9 - Effects of improved crop varieties on agricultural productivity of irrigated pasture. Panel a shows status quo productivity of pasture over the historical period (1980-2010) and the two future periods: 2030-2060 and 2060-2090. Panel b demonstrates how improved pasture varieties affect yield. Panel c shows how probability of bad years changes with new crop varieties, a low yield year in this study is defined as years with productivity less than 60% of fully irrigated condition. Panel d shows the relationship between average yield and standard deviations assuming an unchanged status quo and improved crop varieties. In this figure, the interquartile range in the boxplots is 50% (lower and upper quartile limits are 25% and 75%, respectively). The middle line in the boxplot represents median, and the whiskers can span to 1.5 times the upper and lower interquartile ranges. Outliers (points) in this figure are numbers outside the whiskers. Also, in Panel c, n=10 30-year average values of simulated yield calculated for five GCMs over two future periods.

Supplementary Figure 10

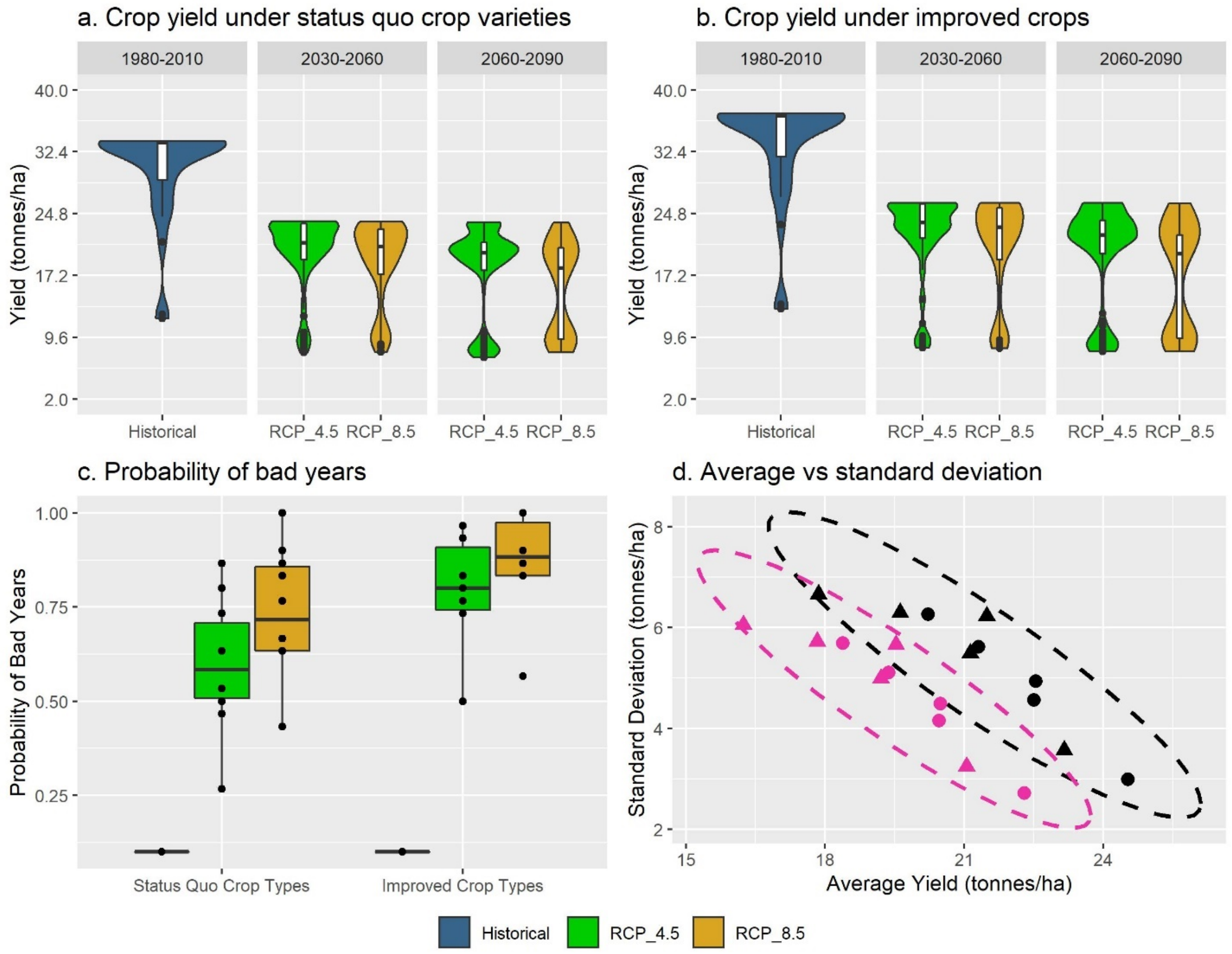

Supplementary Figure 10 - Effects of improved crop varieties on agricultural productivity of irrigated apple. Panel a shows status quo productivity of apple over the historical period (1980-2010) and the two future periods: 2030-2060 and 2060-2090. Panel b demonstrates how improved apple varieties affect yield. Panel c shows how probability of bad years changes with new crop varieties, a low yield year in this study is defined as years with productivity less than 60% of fully irrigated condition. Panel d shows the relationship between average yield and standard deviations assuming an unchanged status quo and improved crop varieties. In this figure, the interquartile range in the boxplots is 50% (lower and upper quartile limits are 25% and 75%, respectively). The middle line in the boxplot represents median, and the whiskers can span to 1.5 times the upper and lower interquartile ranges. Outliers (points) in this figure are numbers outside the whiskers. Also, in Panel c, n=10 30-year average values of simulated yield calculated for five GCMs over two future periods.

Supplementary Figure 11

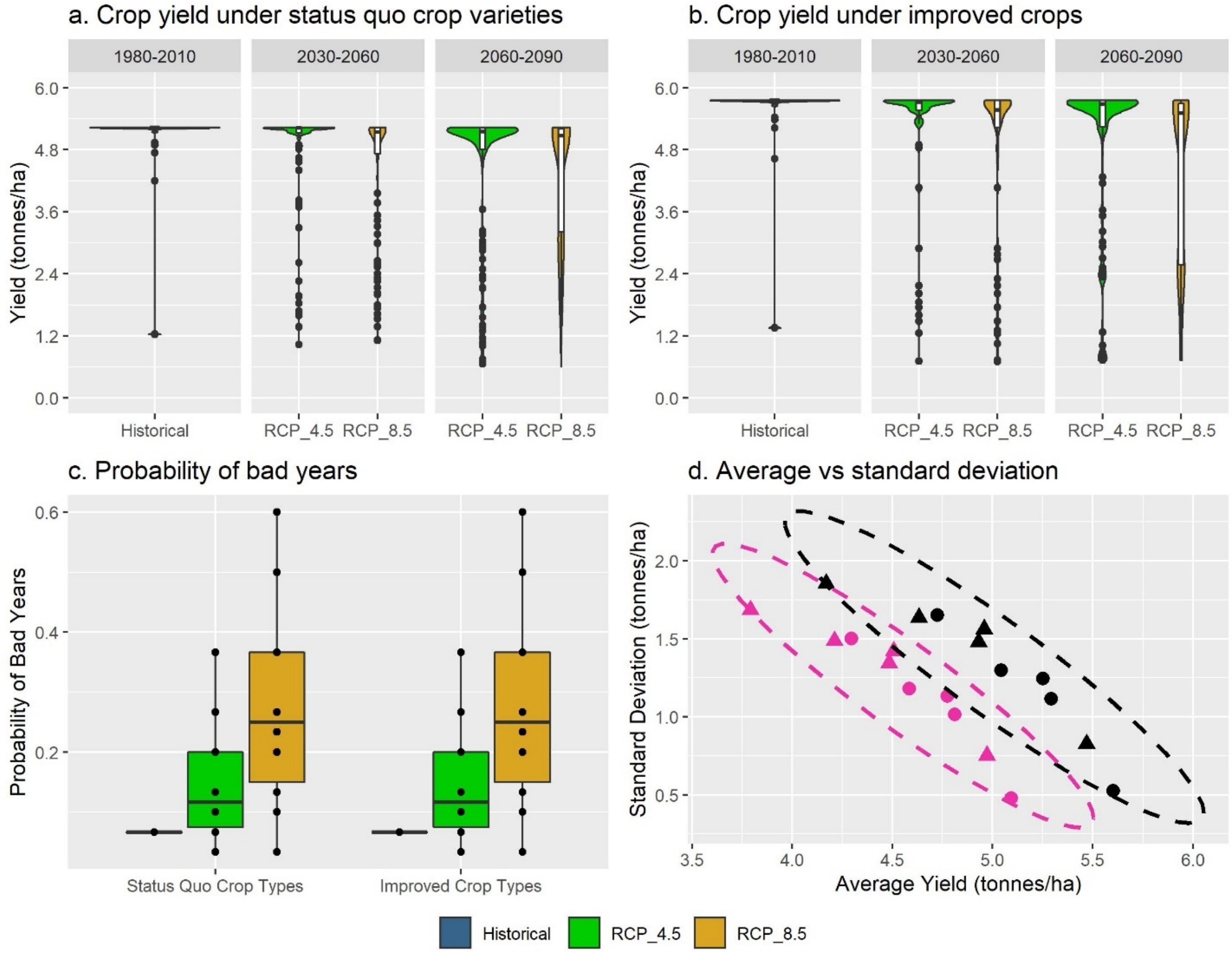

Supplementary Figure 11 - Effects of improved crop varieties on agricultural productivity of irrigated cherries. Panel a shows status quo productivity of cherries over the historical period (1980-2010) and the two future periods: 2030-2060 and 2060-2090. Panel b demonstrates how improved cherries varieties affect yield. Panel c shows how probability of bad years changes with new crop varieties, a low yield year in this study is defined as years with productivity less than 60% of fully irrigated condition. Panel d shows the relationship between average yield and standard deviations assuming an unchanged status quo and improved crop varieties. In this figure, the interquartile range in the boxplots is 50% (lower and upper quartile limits are 25% and 75%, respectively). The middle line in the boxplot represents median, and the whiskers can span to 1.5 times the upper and lower interquartile ranges. Outliers (points) in this figure are numbers outside the whiskers. Also, in Panel c, n=10 30-year average values of simulated yield calculated for five GCMs over two future periods.

Supplementary Figure 12

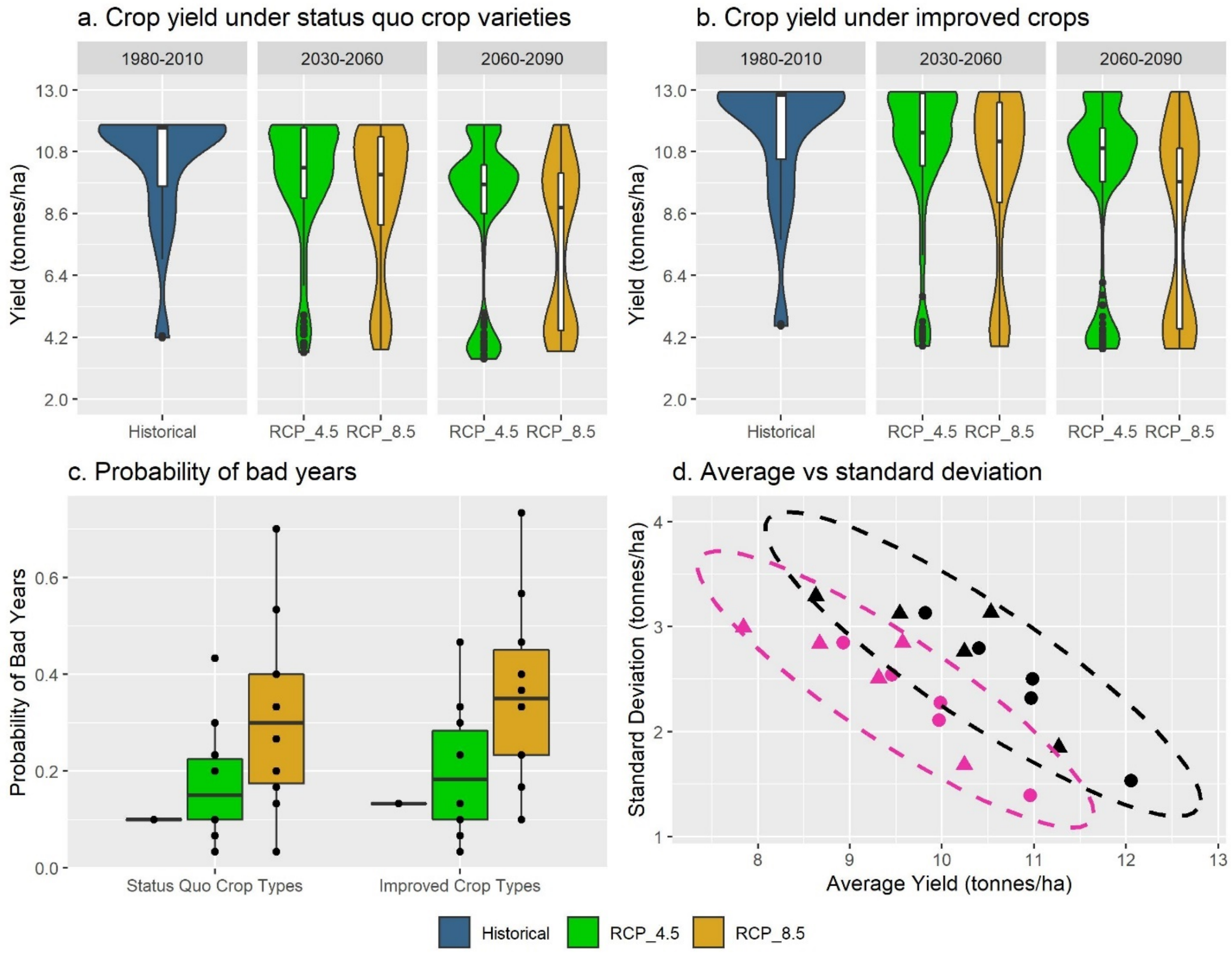

Supplementary Figure 12 - Effects of improved crop varieties on agricultural productivity of irrigated pear. Panel a shows status quo productivity of pear over the historical period (1980-2010) and the two future periods: 2030-2060 and 2060-2090. Panel b demonstrates how improved pear varieties affect yield. Panel c shows how probability of bad years changes with new crop varieties, a low yield year in this study is defined as years with productivity less than 60% of fully irrigated condition. Panel d shows the relationship between average yield and standard deviations assuming an unchanged status quo and improved crop varieties. In this figure, the interquartile range in the boxplots is 50% (lower and upper quartile limits are 25% and 75%, respectively). The middle line in the boxplot represents median, and the whiskers can span to 1.5 times the upper and lower interquartile ranges. Outliers (points) in this figure are numbers outside the whiskers. Also, in Panel c, n=10 30-year average values of simulated yield calculated for five GCMs over two future periods.

Supplementary Figure 13

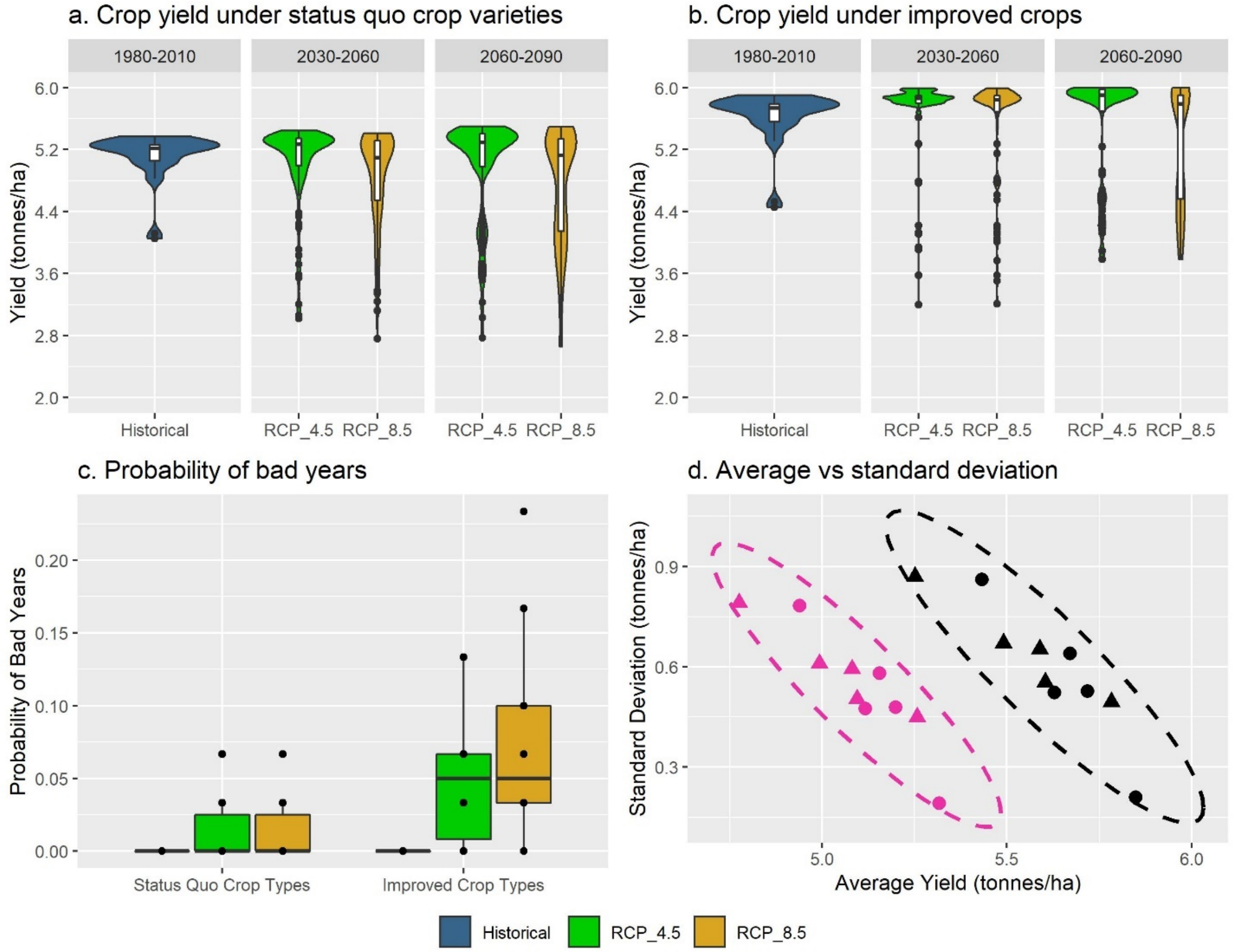

Supplementary Figure 13 - Effects of improved crop varieties on agricultural productivity of irrigated Grapes. Panel a shows status quo productivity of grapes over the historical period (1980-2010) and the two future periods: 2030-2060 and 2060-2090. Panel b demonstrates how improved grapes varieties affect yield. Panel c shows how probability of bad years changes with new crop varieties, a low yield year in this study is defined as years with productivity less than 60% of fully irrigated condition. Panel d shows the relationship between average yield and standard deviations assuming an unchanged status quo and improved crop varieties. In this figure, the interquartile range in the boxplots is 50% (lower and upper quartile limits are 25% and 75%, respectively). The middle line in the boxplot represents median, and the whiskers can span to 1.5 times the upper and lower interquartile ranges. Outliers (points) in this figure are numbers outside the whiskers. Also, in Panel c, n=10 30-year average values of simulated yield calculated for five GCMs over two future periods.

Supplementary Figure 14

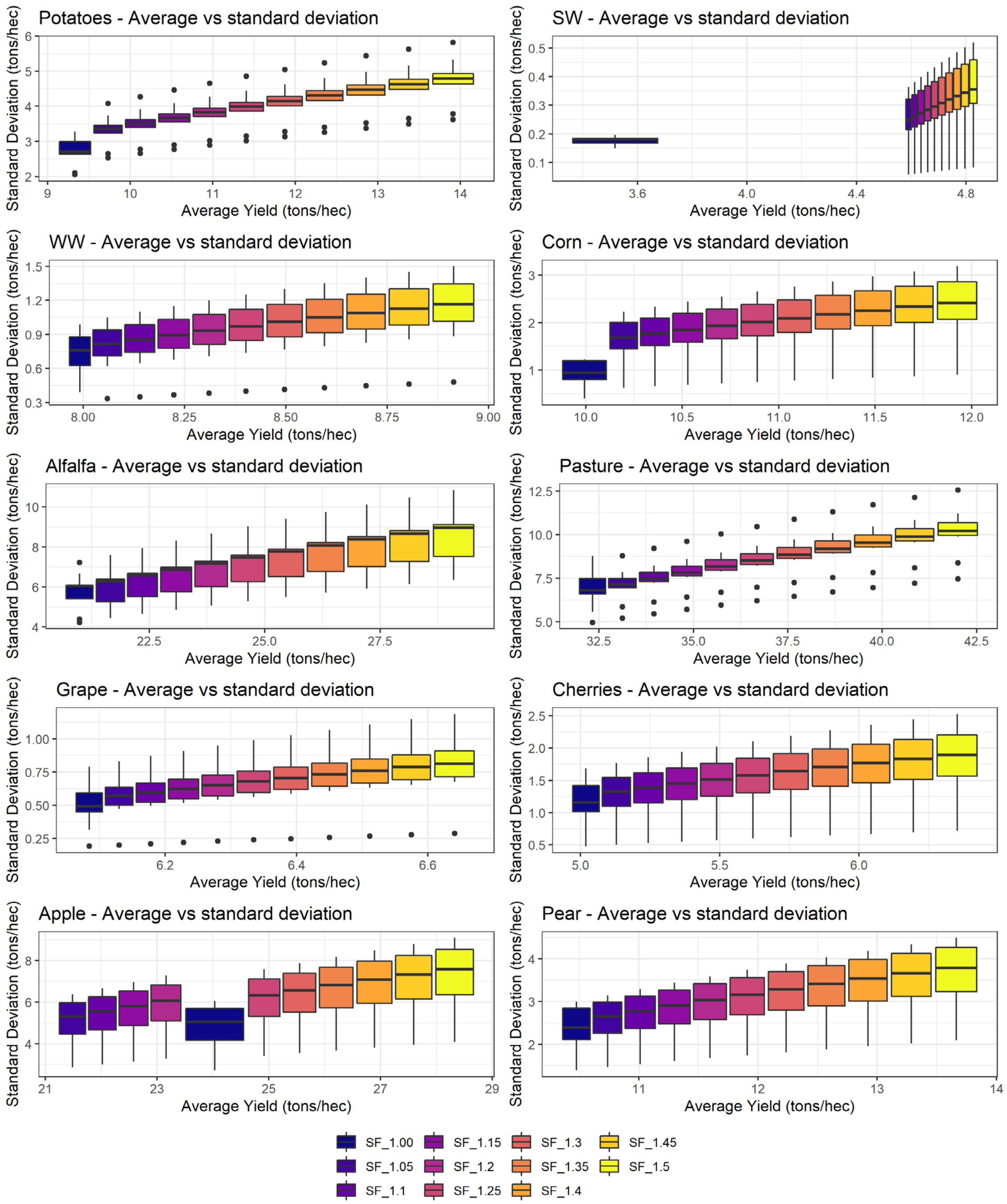

Supplementary Figure 14 - Average and standard deviation of yield under different yield improvement scaling factors (SFs). The boxplots show variability among five GCMs and two RCPs scenarios during two time periods considered in this study (2030-2060 and 2060-2090). In addition to the scaling factors, productivity of annual crops (for scaling factor>1.00) have been adjusted to match with 1990-2000 period. In this figure, the interquartile range in the boxplots is 50% (lower and upper quartile limits are 25% and 75%, respectively). The middle line in the boxplot represents median, and the whiskers can span to 1.5 times the upper and lower interquartile ranges. Outliers (points) in this figure are numbers outside the whiskers. Also, in Panel c, n=10 30-year average values of simulated yield calculated for five GCMs over two future periods.

Supplementary Tables

Supplementary Tables 1

Supplementary Tables 1 - Key bioclimatic thresholds and coefficients used in the VIC-CropSyst model.

	Alfalfa	Spring Wheat	Winter Wheat	Corn	Potato
Early growth temperature limit (°C)	8	8	8	12	8
Cutoff temperature (°C)	25	25	25	30	25
Max temperature (°C)	25	25	25	30	25
Inducement temperature (°C)	5	5	5	5	5
Stomatal closure leaf water potential (m)	-1300	-1300	-1300	-1100	-800
Wilting leaf water potential (m)	-2000	-2000	-2000	-1600	-1200
Root sensitivity water stress Coefficient ([])	0.2	0.2	0.2	0.2	0.3

Supplementary Tables 2

Supplementary Tables 2 - Changes in mean annual agricultural productivity (tonnes/hectare) under different climatic and crop variety scenarios.

Crop Type	Status quo Scenarios			Improved crop variety scenario		
	Historical	RCP_4.5	RCP_8.5	Historical	RCP_4.5	RCP_8.5
Potatoes	11.24	9.12	8.13	12.34	10.41	9.32
Spring Wheat	3.74	3.39	3.41	4.17	4.11	4.04
Winter Wheat	6.77	6.99	6.84	7.53	7.25	7.02
Corn	9.94	7.90	7.41	10.79	10.24	9.76
Alfalfa	21.19	22.38	20.54	23.45	26.40	23.83
Pasture	32.24	29.19	27.33	35.71	32.84	30.44
Grapes	5.12	5.05	4.87	5.60	5.66	5.54
Cherries	5.11	5.01	4.73	5.65	5.39	4.99
Apple	9.91	9.37	8.69	10.97	10.32	9.56
Pear	10.32	9.73	9.02	11.42	10.78	9.97

Supplementary Tables 3

Supplementary Tables 3 - Change in standard deviation (tonnes/hectare) of agricultural productivity under different climatic and crop variety scenarios.

Crop Type	Status quo crop variety			Improved crop variety		
	Historical	RCP_4.5	RCP_8.5	Historical	RCP_4.5	RCP_8.5
Potatoes	3.11	2.19	2.28	3.33	3.33	3.47
Spring Wheat	0.16	0.12	0.13	0.15	0.25	0.33
Winter Wheat	0.63	0.54	0.67	0.56	0.75	0.91
Corn	1.21	0.71	0.85	1.11	1.56	1.93
Alfalfa	7.11	5.89	5.17	7.76	8.22	7.05
Pasture	8.71	5.73	5.41	9.19	7.34	7.54
Grapes	0.31	0.41	0.45	0.38	0.55	0.65
Cherries	1.13	0.87	1.02	1.17	1.32	1.65
Apple	2.03	1.68	1.91	2.15	2.32	2.69
Pear	2.28	1.81	2.05	2.41	2.50	2.88

Supplementary Notes

Supplementary Notes 1: Strategies to Address Mean-Varibility Tradeoffs in Yield

Two broad categories of adapatation (Supplementary Fig. 2) can affect yield variability tradeoffs. The two categories are (1) improvement in water institutions and infrastructures and (2) improvement in crop potential productivity. Section 6 in the main body of the manuscript provides a more comprehensive description of these changes. In summary, improvements in potential yield strongly shape average annual yield and water system constraints can lead to increased volatility for food production and revenue in snow-dominated systems. However, when improvements in water systems and crop varieties simoultaneously happen a more sustaianble agricultural productivity may emerge on the horizon.

Supplementary Notes 2: Key Bioclimatic Thresholds in VIC-CropSyst

The VIC-CropSyst coupled simulation framework combines the Variable Infiltration Capacity (VIC) hydrological model (to simulate the regional water and energy cycle) and the Cropping Systems Simulation Model (CropSyst, to simulate agricultural and farm-level biogeochemical processes). In the coupled model, CropSyst simulates crop-related processes such as growth, transpiration, biomass, and yield production. CropSyst accounts for several bioclimatic thresholds (Supplementary Tables 1); for example, crops in CropSyst respond to a minimum base temperature. Temperatures higher than the base temperature activate all of the biophysical processes of the crop. CropSyst also takes into account a crop-specific temperature threshold after which it will reduce its growth rate. CropSyst also has a maximum temperature at which all crop-related processes stop. The impacts of temperature on crop productivity also vary by growing period in the model. Crops in CropSyst respond to water deficiencies. Lower water availability slows down crop growth and development and can eventually lead to a crop's death. Although CropSyst also simulates the impacts of nutrient deficiency on crop processes, this aspect of the model is not used in this study. CropSyst captures the impacts of future projected higher atmospheric CO2 on growth, production of yield, and biomass. CropSyst also simulates root development and distributes water extraction from different soil layers based on water availability and root density.

Supplementary Notes 3: Improved Crop Varieties

Improvement in crop varieties have been suggested as one of the main pathways to improve the productivity of agriculture and global food security[1]. A 150% increase in average yield of wheat, corn and rice since 1960 has been regarded as a primary driver of improved global food security[2]. In this study, we explore the yield mean-variability tradeoff in snow-dominated regions by assuming that the growing period of annual crops (e.g., wheat, corn, and potatoes) will increase in the future to respond to the climate change-induced accelerated growing period[1,3]. We also assume that a second adaptation pathway has succeeded in yielding a 10% improvement in the average yield of all crop types (as represented by increasing the harvest indices for our modeled crops). In reality, a 10% improvement average annual yield can be realized through a myriad of adaptation pathways[2,4–7]. For example, yields can be improved by improving the resistance of crops to biotic stressors such as fungi, bacteria, oomycetes, and various other pests or diseases. Also, varieties with higher abiotic resistances can lead us toward the 10% improvement goal. Abiotic stressors can include sunlight, toxic soil minerals, salinity, droughts, excess water, heatwaves, and freezing events. The physical characteristics of crops such as root size and crop heights can also be adapted, and this can have direct and indirect implications for crop yield. Finally, varieties with longer growing periods can increase the crop opportunity for biomass accumulation; by contrast, in certain situations, crops with shorter growing periods can reduce the chance of water and heat stress at the end of the season[5]. Adaptations to crop growth rate can also reduce the chance of extreme events during crop-sensitive periods. In this study, although we do not specify the specific pathways that are employed to improve the average yield by 10%; however, this result is well within the scope of the published mechanisms[2,8,9] for improving expected annual yields.

In other words, in this study, to construct the improved crop varieties, we use the following two assumptions. (1) For annual crops (i.e., potatoes, winter wheat, spring wheat, and corn), we assume that the improved crops mimic growth rates and productivity properties of crops during the 1990–2000 period. We also assume that agricultural productivity of these seeds increases by 10%. These improved varieties respond to the impacts of climate change-induced higher temperature on shortening of growing period of annual crops [10–13]. The prolonged varieties provide longer time for photosynthesis and yield accumulations[11]. (2) For multiple-cutting crops (i.e., alfalfa and pasture) and perennial tree fruits (i.e., cherries, apples, pears, and grapes), improved varieties will increase the productivity by 10% compared to figures from the historical periods. We do not explicitly explore which adaptation strategies can lead to that 10% improvement here. However, for the case of cereal crops, we explicitly assume that the crop-growing period will be longer in the modified varieties. The reason is that cereal crops are likely to experience a temperature-driven expedited growing period in the future[3,14]. Faster maturity leads to less

opportunity for accumulation of biomass and yield[15]. Many past studies have suggested that the food security of the world would be compromised if we do not immediately react to this[1,3,6].

Supplementary Notes 4: Impacts of Climate Change on Actual Yield Loss During Drought Years

Supplementary Figure 3 shows that climate change can reduce yield loss during drought years. We define yield loss as the difference between fully irrigated yield and deficit-irrigated yield. To recap, irrigation water in the YRB comes from a regional surface-water system; this system is snow dominated and controlled by an interconnected and complicated web of precipitation, snow-melting process, dam operation, and water rights. The method section in the main body of the paper provides more information. Therefore, deficit irrigation in our simulation is triggered by regional water availability in the YRB.

We show that yield loss mainly decreases due to a reduction in potential yield (under fully irrigated conditions). Increases in minimum yield also tighten the gap between fully irrigated and deficit-irrigated conditions. The Supplementary Notes 5 shows how various crops respond to climate change and why minimum and maximum yields change. More information on this subject can be found in the main body of the manuscript (Section 3).

Supplementary Notes 5: Crop Maximum and Minimum Yields

Supplementary Figure 3 shows that climate change can reduce the potential productivity of many crop types such as spring wheat, potatoes, and corn. The main reason behind the productivity reduction is an acceleration of crop growth due to climate-change-induced higher temperature. In other words, warming shortens the growing period, thereby decreasing the amount of time for photosynthesis and biomass accumulation. However, winter wheat responds slightly differently and shows almost no change in crop growth. This slightly different behavior is explained by the fact that winter wheat is a winter crop; its dormancy period and early season times give the plants more time to photosynthesize. Our results also indicate that perennial tree fruits will not be significantly affected by climate change, because the length of their growing period will remain the same. The only crop group that can experience an increase in maximum yield is multiple-cutting crops (e.g., alfalfa). Higher temperatures do not lead to a faster arrival of the end of the season; instead, higher temperatures can increase the number of cuttings, thereby improving productivity. This is different from the situation for annual crops such as potatoes and wheat.

However, minimum yield will increase in the future. This trend is consistent among all the crop types. Two main factors that control improvement in the minimum yield are higher CO_2, which intensifies crop

photosynthesis, and an overall increase in precipitation. In non-irrigated agriculture, the length of the growing season is not typically a limiting factor, and crops can usually reach their attainable growth conditions with limited water.

Supplementary Notes 6: Sensitivity to Crop Improvement Assumptions

To further evaluate our yield improvement hypothesis (10% improvement in the potential yield), we explored the sensitivity of our results to different improvement scaling factors. The results suggest that for all scenarios, and for all crop types, improvement in potential productivity lead to higher production volatility (Supplementary Figure 14). In the sensitivity analysis of the annual crops we used the crop response curves of our benchmark historical period (1990-2000) which further deviates the average yield from the baseline scenario.

Supplementary References

1. Atlin, G. N., Cairns, J. E. & Das, B. Rapid breeding and varietal replacement are critical to adaptation of cropping systems in the developing world to climate change. *Glob. Food Secur.* **12**, 31–37 (2017).
2. Chapter 18 - Crop Variety Improvements. in *Sustainable Food and Agriculture* (eds. Campanhola, C. & Pandey, S.) 205–207 (Academic Press, 2019). doi:10.1016/B978-0-12-812134-4.00018-2.
3. Challinor, A. J., Koehler, A.-K., Ramirez-Villegas, J., Whitfield, S. & Das, B. Current warming will reduce yields unless maize breeding and seed systems adapt immediately. *Nat. Clim. Change* **6**, 954–958 (2016).
4. Etten, J. van *et al.* Crop variety management for climate adaptation supported by citizen science. *Proc. Natl. Acad. Sci.* **116**, 4194–4199 (2019).
5. Fahad, S. *et al.* Crop Production under Drought and Heat Stress: Plant Responses and Management Options. *Front. Plant Sci.* **8**, (2017).
6. Korres, N. E. *et al.* Cultivars to face climate change effects on crops and weeds: a review. *Agron. Sustain. Dev.* **36**, 12 (2016).
7. Cairns, J. E. & Prasanna, B. Developing and deploying climate-resilient maize varieties in the developing world. *Curr. Opin. Plant Biol.* **45**, 226–230 (2018).
8. Howden, S. M. *et al.* Adapting agriculture to climate change. *Proc. Natl. Acad. Sci.* **104**, 19691–19696 (2007).
9. Lobell, D. B. & Gourdji, S. M. The Influence of Climate Change on Global Crop Productivity. *Plant Physiol.* **160**, 1686–1697 (2012).
10. Bai, H., Tao, F., Xiao, D., Liu, F. & Zhang, H. Attribution of yield change for rice-wheat rotation system in China to climate change, cultivars and agronomic management in the past three decades. *Clim. Change* **135**, 539–553 (2016).
11. Rezaei, E. E., Siebert, S., Hüging, H. & Ewert, F. Climate change effect on wheat phenology depends on cultivar change. *Sci. Rep.* **8**, 1–10 (2018).

12. Xiao, D. *et al.* Impact of warming climate and cultivar change on maize phenology in the last three decades in North China Plain. *Theor. Appl. Climatol.* **124**, 653–661 (2016).

13. Asseng, S. *et al.* Rising temperatures reduce global wheat production. *Nat. Clim. Change* **5**, 143–147 (2015).

14. Schauberger, B. *et al.* Consistent negative response of US crops to high temperatures in observations and crop models. *Nat. Commun.* **8**, 13931 (2017).

15. Zhao, C. *et al.* Plausible rice yield losses under future climate warming. *Nat. Plants* **3**, 1–5 (2016).
