# Supplementary material for: Water rights shape crop yield and revenue volatility tradeoffs for adaptation in snow dependent systems
Source: Nat Commun. 2020 Jul 10;11:3473. doi: 10.1038/s41467-020-17219-z (PMC7351950; doi:10.1038/s41467-020-17219-z)
Supplement: Supplementary file 2 — Reporting Summary [file 41467_2020_17219_MOESM2_ESM.pdf]

nature research

Corresponding author(s): Keyvan Malek
Last updated by author(s): 2020/06/08

Reporting Summary

Nature Research wishes to improve the reproducibility of the work that we publish. This form provides structure for consistency and transparency in reporting. For further information on Nature Research policies, see Authors & Referees and the Editorial Policy Checklist .

Statistics

For all statistical analyses, confirm that the following items are present in the figure legend, table legend, main text, or Methods section.

n/a	Confirmed	
x		The exact sample size (*n*) for each experimental group/condition, given as a discrete number and unit of measurement
x		A statement on whether measurements were taken from distinct samples or whether the same sample was measured repeatedly
x		The statistical test(s) used AND whether they are one- or two-sided<br>*Only common tests should be described solely by name; describe more complex techniques in the Methods section.*
x		A description of all covariates tested
x		A description of any assumptions or corrections, such as tests of normality and adjustment for multiple comparisons
	x	A full description of the statistical parameters including central tendency (e.g. means) or other basic estimates (e.g. regression coefficient) AND variation (e.g. standard deviation) or associated estimates of uncertainty (e.g. confidence intervals)
x		For null hypothesis testing, the test statistic (e.g. *F*, *t*, *r*) with confidence intervals, effect sizes, degrees of freedom and *P* value noted<br>*Give P values as exact values whenever suitable.*
x		For Bayesian analysis, information on the choice of priors and Markov chain Monte Carlo settings
x		For hierarchical and complex designs, identification of the appropriate level for tests and full reporting of outcomes
x		Estimates of effect sizes (e.g. Cohen's *d*, Pearson's *r*), indicating how they were calculated

Our web collection on statistics for biologists contains articles on many of the points above.

Software and code

Policy information about availability of computer code

Data collection	We used VIC-CropSyst-v2 to simulate agro-hydrologic processes, the model will be made available upon email request to Keyvan Malek (km663@cornell.edu) and Jennifer Adam (jcadam@wsu.edu). We used Yakima RiverWare (YAK-RW) which is a commercialized software to simulate the river system processes. We provide this information as well as inputs (and outputs) to the YAK-RW model in our DOI repository.
Data analysis	Our GitHub page and its Digital Object Identifier (DOI: 10.5281/zenodo.3743865) provides all our post-processing codes and data.

For manuscripts utilizing custom algorithms or software that are central to the research but not yet described in published literature, software must be made available to editors/reviewers. We strongly encourage code deposition in a community repository (e.g. GitHub). See the Nature Research guidelines for submitting code & software for further information.

Data

Policy information about availability of data

All manuscripts must include a data availability statement. This statement should provide the following information, where applicable:

- Accession codes, unique identifiers, or web links for publicly available datasets
- A list of figures that have associated raw data
- A description of any restrictions on data availability

Although the supplemental materials provide a comprehensive description of the data and models used in this study, readers can refer to the GitHub repository of this paper to access the model, data and our post-processing scripts (DOI: 10.5281/zenodo.3743865).

Field-specific reporting

Please select the one below that is the best fit for your research. If you are not sure, read the appropriate sections before making your selection.

- [ ] Life sciences
- [ ] Behavioural & social sciences
- [x] Ecological, evolutionary & environmental sciences

For a reference copy of the document with all sections, see nature.com/documents/nr-reporting-summary-flat.pdf

Ecological, evolutionary & environmental sciences study design

All studies must disclose on these points even when the disclosure is negative.

Study description	We explored the impacts of climate change on water resources and agricultural productivity of an agriculturally important area in the US Pacific Northwest
Research sample	In this study, we used a couple agricultural-hydrologic model called VIC-CropSyst. The model uses a gridded meteorological data (i.e., GFDL-ESM2G, HadGEM2-ES3, HadGEM2-CC365, INMCM4, and CanESM2), soil inputs (STASGO), and crop and vegetative information (Malek et al 2018).
Sampling strategy	We selected the above 5 GCMs from a broader list of 20 GCMs. The selection as described in manuscript was done to represents a wide range of possible temperature and precipitation changes in future. We used all of our model outputs to generate the results of our paper
Data collection	We conducted our simulations and a part of our post-processing on computer clusters
Timing and spatial scale	Our preliminary simulations were conducted in 2018. However, we conducted most of our simulations and our data analysis in 2019.
Data exclusions	No data were excluded from our study
Reproducibility	Our computational experiment was tested and is reproducible
Randomization	This is not relevant to our study, we do not allocate samples to groups.
Blinding	This is not relevant to our study.

Did the study involve field work? [ ] Yes [x] No

Reporting for specific materials, systems and methods

We require information from authors about some types of materials, experimental systems and methods used in many studies. Here, indicate whether each material, system or method listed is relevant to your study. If you are not sure if a list item applies to your research, read the appropriate section before selecting a response.

Materials & experimental systems

n/a	Involved in the study	
[x]	[ ]	Antibodies
[x]	[ ]	Eukaryotic cell lines
[x]	[ ]	Palaeontology
[x]	[ ]	Animals and other organisms
[x]	[ ]	Human research participants
[x]	[ ]	Clinical data

Methods

n/a	Involved in the study	
[x]	[ ]	ChIP-seq
[x]	[ ]	Flow cytometry
[x]	[ ]	MRI-based neuroimaging
